# A Web-Based Adolescent Positive Psychology Program in Schools: Randomized Controlled Trial

**DOI:** 10.2196/jmir.4329

**Published:** 2015-07-28

**Authors:** Rowan Burckhardt, Vijaya Manicavasagar, Philip J Batterham, Leonie M Miller, Elizabeth Talbot, Alistair Lum

**Affiliations:** ^1^ University of NSW and the Black Dog Institute Randwick Australia; ^2^ National Institute for Mental Health Research, The Australian National University Canberra Australia; ^3^ School of Psychology, University of Wollongong Wollongong Australia

**Keywords:** adolescent, Internet, early medical intervention, randomized controlled trial

## Abstract

**Background:**

Adolescent mental health is characterized by relatively high rates of psychiatric disorders and low levels of help-seeking behaviors. Existing mental health programs aimed at addressing these issues in adolescents have repeated inconsistent results. Such programs have generally been based on techniques derived from cognitive behavioral therapy, which may not be ideally suited to early intervention among adolescent samples. Positive psychology, which seeks to improve well-being rather than alleviate psychological symptoms, offers an alternative approach. A previous community study of adolescents found that informal engagement in an online positive psychology program for up to 6 weeks yielded significant improvements in both well-being and depression symptoms. However, this approach had not been trialed among adolescents in a structured format and within a school setting.

**Objective:**

This study examines the feasibility of an online school-based positive psychology program delivered in a structured format over a 6-week period utilizing a workbook to guide students through website content and interactive exercises.

**Methods:**

Students from four high schools were randomly allocated by classroom to either the positive psychology condition, "Bite Back", or the control condition. The Bite Back condition consisted of positive psychology exercises and information, while the control condition used a series of non-psychology entertainment websites. Both interventions were delivered online for 6 hours over a period of 4-6 weeks during class time. Symptom measures and measures of well-being/flourishing and life satisfaction were administered at baseline and post intervention.

**Results:**

Data were analyzed using multilevel linear modeling. Both conditions demonstrated reductions in depression, stress, and total symptom scores without any significant differences between the two conditions. Both the Bite Back and control conditions also demonstrated significant improvements in life satisfaction scores post intervention. However, only the control condition demonstrated significant increases in flourishing scores post intervention.

**Conclusions:**

Results suggest that a structured online positive psychology program administered within the school curriculum was not effective when compared to the control condition. The limitations of online program delivery in school settings including logistic considerations are also relevant to the contradictory findings of this study.

**Trial Registration:**

Australian New Zealand Clinical Trials Registry: ACTRN1261200057831; https://www.anzctr.org.au/Trial/Registration/TrialReview.aspx?id=362489 (Archived by Webcite at http://www.webcitation.org/6NXmjwfAy).

## Introduction

Epidemiological data indicate that 1 in 4 young people in Australia between the ages of 16-24 years have experienced at least one mental disorder in the preceding year and that young people have the highest rates of mental disorders compared to any other age group [1]. It has been estimated that of those aged 16-24 years experiencing mental health issues, 77% did not access any medical or professional services in the preceding 12 months and that they account for almost 30% of the total mental health burden in Australia [2,3]. This is further aggravated by their relative lack of skills in dealing with life stresses and emotional distress compared to adults [4].

A number of early intervention programs have been developed to address skills deficits and to build resilience in young people. Most of these programs have used cognitive behavioral therapy techniques with varying degress of success [5]. This suggests that researchers need to look further afield for effective prevention approaches to youth mental health such as the building of skills and strategies to enhance personal strengths [6].

In this regard, positive psychology—the scientific study of happiness, well-being, and flourishing—may offer an alternative to conventional prevention programs for youth. Positive psychology can be conceptualized as a range of behavioral, cognitive, and emotional domains that are important for improving well-being. These include developing healthy social relationships, becoming more optimistic, finding “meaning”, and practicing mindfulness. Evaluations of non-school-based programs have reported on the benefits of engaging in positive psychology exercises. For example, interventions that increase hope have been shown to predict lower illicit substance use; lower levels of depression, anxiety, and hostility; fewer behavioral problems; and higher academic performance in adolescents [7,8].

Manicavasagar et al conducted a feasibility study of a community-based online positive psychological program, “Bite Back”, for adolescents aged 12-18 years [9,10]. Bite Back consists of information and interactive activities relating to nine domains: gratitude, optimism, flow, meaning, hope, mindfulness, character strengths, healthy lifestyle, and positive relationships. Participants in both the Bite Back condition and a control condition were encouraged to regularly use their respective websites over a 6-week period. Symptom measures of depression, anxiety and stress, and well-being were compared within and between conditions and at baseline and post intervention. At the start of the intervention, participants reported low levels of psychopathology, as was expected in a non-clinical sample. Results demonstrated significant decreases in symptom scores and increased scores on well-being post intervention for the Bite Back relative to the control condition. It was reported that when participants with high levels of engagement were examined in isolation, the findings were even stronger suggesting that increased usage was related to greater benefits. Furthermore, qualitative analyses indicated that Bite Back was well received by participants allocated to this condition. It is notable that participants using this online positive psychology program could freely engage with the material at their own pace and in their own time. However, the open-access online format of this program meant that the researchers were unable to examine how participants navigated the website and whether they were exposed to the full range of material and information about positive psychology.

A literature search did not uncover any previously evaluated computer-based school-delivered positive psychology interventions. However, group-format school-based positive psychology interventions, such as the Positive Psychology Program and Wellness-Promotion Intervention, consist of lesson-based modules pertaining to specific topics and skills delivered over the intervention period [11-16]. It was therefore deemed timely to evaluate Bite Back in a structured lesson-based format that was appropriate for delivery within a school environment. This study explores the feasibility of implementing a structured workbook-guided version of Bite Back in senior schools as part of the schools’ curriculum. It was expected that the benefits found in our previous study would be extended to this student population, specifically, that participants would improve on measures of well-being and report decreased symptoms scores following the intervention compared to a control condition.

## Methods

### Participants

Four Australian high schools agreed to participate in the study: two Anglican girls’ schools (referred to as “Girls School A” and “Girls School B”), a Catholic boys’ school (referred to as “Boys School”), and a Jewish co-educational school (referred to as “Co-ed School”). The level of religiosity of students within these schools was not formally assessed although they are considered by reputation to be approximately equally based on conversations with staff in the educational system. Students were in Grades 7 through 12, which in the Australian educational system are the final 6 years before being eligible to go to university. These four schools were among the highest in terms of socioeconomic status compared to other schools in Australia. Students were included in the study only if they completed the relevant consent forms. This study was approved by the University of NSW Human Research Ethics Advisory Committee (Ethics Approval number 2011-7-35). See Multimedia Appendix 1 for the CONSORT-EHEALTH checklist [17].

### Interventions

#### The Positive Psychology Condition (Bite Back)

**Figure 1:**
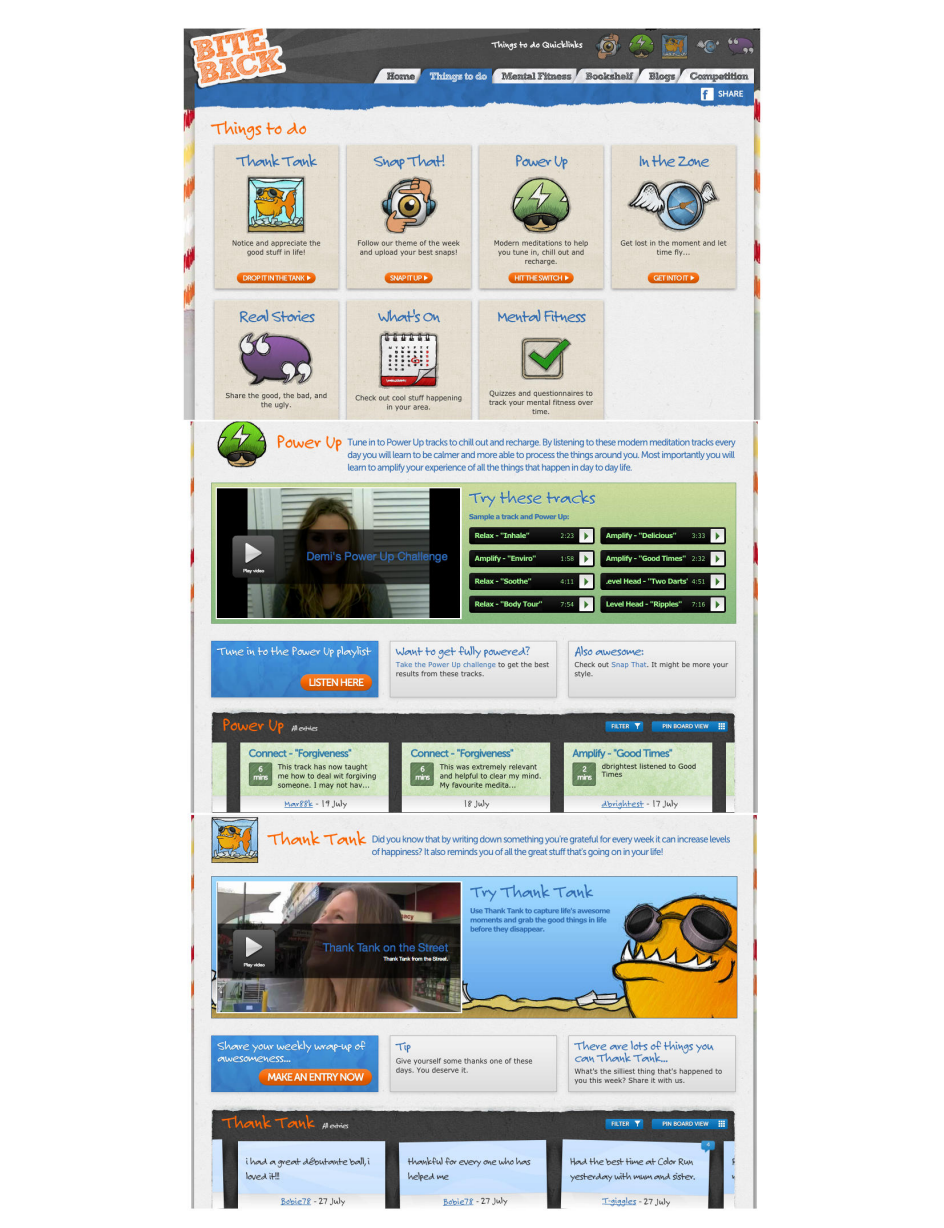
Screenshots from the Bite Back website.

Bite Back was developed by the Black Dog Institute to improve the well-being and happiness of young Australians aged 12-18 years. Key objectives of this program are to encourage young people to work to their full potential, become more fully engaged in all aspects of their lives, and ultimately, to build resilience. The program consists of a range of interactive activities including making gratitude entries, mindfulness meditations, describing personal stories, and a mindfulness exercise involving taking photos. Figure 1 provides screenshots of the website to illustrate key components. Online interactive exercises are designed to encourage generalization of online activities to the “real world”, so that young people benefit from implementing the central tenets of positive psychology into their daily lives. Also included on the site is information about nine positive psychology domains and ways to engage with them outside of the website: Gratitude, Optimism, Flow, Meaning, Hope, Mindfulness, Character Strengths, Healthy Lifestyle, and Positive Relationships. The nine domains were based on research that suggested they were important in improving well-being [18]. Users are able to comment on activities (such as submitted photos or stories by other users) with their anonymous profile thereby enabling participation without fear of stigma or judgment. The website is pre-moderated by website staff to ensure that antisocial or concerning behavior is addressed prior to possible posting on the website. In the rare cases that a posting is either concerning from a mental health perspective or abusive, a senior clinical psychologist advised on how to handle it (eg, send an email suggesting they seek external assistance). Confidentiality of users was ensured by requiring new users to sign up using a pseudonym—no real names are permitted. Users can also choose to post content in private, where they are the only ones who can view it, or they can post it publicly. To ensure security of content and users, Bite Back uses password-protected accounts, encrypted password storage, encrypted login details, and secure external servers. For the purposes of the study, a workbook was developed to guide students through the positive psychology exercises on the website and to pose reflective questions about positive psychology. This ensured that participants had sufficient material to use during the course of the program and encouraged users to engage with several domains within the website. Participants were required to record all comments and answers to questions in their workbooks.

#### The Control Condition

Participants assigned to the control condition used a workbook that was structured in a similar format to that of the Bite Back workbook but with questions and links to non-psychology websites: Australian Broadcasting Corporation, Australian Youth Climate Coalition, the Natural Resource Defense Council, World Wildlife Fund, Wikipedia, Internet Movie Database, and World Youth News. Questions such as “Do you read online news websites in your own time? If yes, which ones?”, “Do you think the news makes a difference to how you live in your daily life? Why?”, and “Write a summary of what the article was about” were designed to encourage users to engage with these websites and record their comments and answers to questions in their workbooks.

### Procedure

Between January and March 2012, 20 schools were invited to participate in this study, of which four agreed. The 20 schools were chosen based on two criteria: physical proximity to the research institute and being non-governmental. The proximity factor was to facilitate the practical running of the study given that trips to the school were necessary and the non-governmental requirement because access to these schools is less onerous and more timely than governmental institutions, which require permission from the state school department that takes over 6 months to obtain. Prior to start of the study, the lead author met with senior school staff and welfare officers to convey the requirements of the study and to instruct teachers in the study methodology. Parental and student self-consent were obtained for all participants under the age of 16 years, and student self-consent only was obtained for participants aged 16 years and over. The grade level designated to participate in the study was chosen by the school, and no exclusion criteria were applied.

Teachers were instructed to explain to students that they were participating in a study designed to examine the way that websites affect how young people view the world. Teachers were also required to provide a 5-minute discussion on this topic prior to the beginning of the study and were asked not to mention that the study included a positive psychology intervention. Teachers were responsible for handing out workbooks, providing students with access to relevant websites, and managing students’ behavior during class time. Teachers were unaware of which classes had been allocated to the intervention or control conditions. Students had no face-to-face contact with any of the researchers prior to, during, or following the study.

Students were randomized by blocks (classes) using an Excel random number generator to allocate them to either the Bite Back or control conditions by an independent researcher not associated with the data collection. Equivalent numbers of students were allocated to each condition across each grade level and for each school (eg, Grade 9 at the Boys School). In cases where there were uneven numbers of blocks, students were assigned to the Bite Back condition. Students completed an online battery of self-report questionnaires twice during class time: pre-intervention and post intervention.

All schools were instructed to use the workbook over a 6-week period. However, two schools (the Boys School and the Co-ed School) completed the workbook over a 4-week period due to class-time constraints. Teachers were told that a total of 6 hours of face-to-face time was required from students over a period of 4-6 weeks. After each session, students in both the control and Bite Back conditions were asked to email completed sections of their workbooks to the researchers. This strategy was employed to encourage engagement and increase compliance. It also served as an indicator of student adherence to the research tasks. Students were advised that their teachers did not have access to their workbooks and completed questionnaires.

### Measures

The Depression, Anxiety, and Stress Scale—Short form (DASS-21) consists of three symptom-based subscales [19]. Each subscale has 7 items that participants respond to on a 4-point Likert scale (0=“not at all” to 3=“most of the time”). Summed scores for each scale range from 0-42 following conversion of scores to match the DASS-42; more severe symptoms are indicated by higher scores. The DASS has also been demonstrated to correlate closely with the Diagnostic and Statistical Manual of Mental Disorders’ anxiety and depressive diagnoses [20]. Although the DASS-21 has been previously used on adolescent samples [21-23], we modified some of the wording to improve comprehension (eg, “I felt down-hearted and blue” was changed to “I felt down”). These changes were approved by the DASS-21 authors and the original meaning of the items remained unchanged. Cronbach alpha was calculated for the sample, which indicated that it was .91 for the depression scale, .79 for anxiety, .81 for stress, and .92 for the total score. The DASS-21 total score was analyzed in addition to subscale scores because of evidence that adolescent psychometrics may not support a three-factor model but rather a single factor [21,23].

Student Life Satisfaction Scale (SLSS) comprises 5 items from the original 7-item SLSS, which are rated on a 7-point Likert scale (1=“strongly disagree” to 7=“strongly agree”) that measures quality of life (eg, “I have what I want in life”, “My life is going well”) [24]. Higher scores indicate a higher subjective satisfaction with life. Cronbach alpha in the current sample was .90.

The Short Warwick-Edinburgh Mental Well-Being Scale (SWEMWBS) is a 7-item measure that assesses positive mental health (well-being) over the past 2 weeks rated on a 5-point scale (1=“none of the time” to 5=“all of the time”) [25]. Psychometric data of the measure on the original WEMWBS for adolescents indicated satisfactory to high internal consistency (*r=*.87), and the short version has acceptable test-retest reliability (*r=*.66, 95% CI 0.59-0.72) [25,26]. In our sample, Cronbach alpha was .82.

### Statistical Analysis

Data were analyzed using SPSS version 22 [27]. Independent samples *t* tests were used to examine group differences at baseline. Multilevel linear modeling was used to examine the effectiveness of the intervention in reducing depression, anxiety and stress, and increasing life satisfaction and well-being. This type of model represents an intention-to-treat analysis under the missing-at-random assumption. Specifically, the effect of time (posttest vs pretest) was estimated using a repeated-measures mixed-effects model, which also accounted for clustering of students within schools using a random intercept for each school. An unstructured variance-covariance structure was assumed for both the repeated effect of time and the random effect of school. Degrees of freedom were estimated using Satterthwaite’s correction [28].

## Results

### Flow of Participants and Participant Characteristics

**Figure 2:**
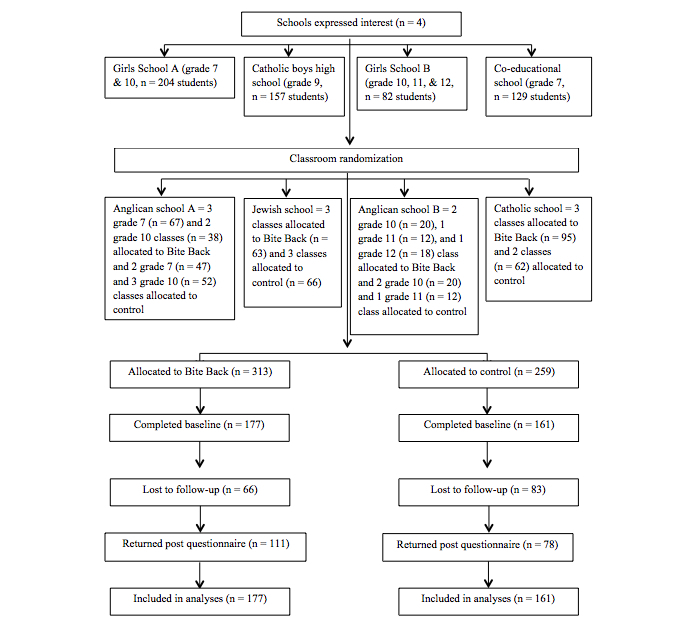
CONSORT diagram of participants.

Figure 2 demonstrates the flow of participants during the course of the study. Four schools provided a total of 572 students across grades 7-12, who were randomly allocated through classroom blocks to either the control or Bite Back conditions. Of those allocated, 338 students completed a battery of online questionnaires at baseline and were considered to be enrolled in the study. From the 234 students who did not complete the baseline questionnaire, 110 encountered technical difficulties that prevented them from being able to access the questionnaire successfully. The other 124 either chose not to fill out the questionnaire or were absent. Of the four schools that were enrolled, one withdrew from the study (Girls School B) and one ceased to participate because the teacher involved in its implementation went on extended leave (Co-ed School). Baseline characteristics of participants are presented in Table 1.

A series of independent samples *t* tests examined baseline differences on questionnaire scores between the two conditions (control versus Bite Back groups across the entire sample). No significant differences were observed between the groups for the depression subscale, *P*=.40; the anxiety subscale, *P*=.60; the stress subscale, *P*=.11; the DASS-total score, *P*=.25; or the SLSS, *P*=.15. A significant difference was observed on the SWEMWBS (*t*_332_=-4.1, *P*<.001) indicating that the Bite Back group began the study with higher levels of well-being (mean 18.2) than the control group (mean 16.7). The size of the difference was moderate using the guidelines proposed by Cohen [29] (eta squared=0.05).

**Table 1:** Baseline characteristics of participants in the school study.

		Girls A		Boys		Girls B		Co-ed		
		Bite Back (n=94)	Control (n=90)	Bite Back (n=72)	Control (n=54)	Bite Back (n=8)	Control (n=5)	Bite Back (n=1)		Control (n=12)
Grades		7 and 10		9		10 and 11		7		
Female, n (%)		184 (100)		0 (0)		13 (100)		3 (23)		
Age in years, mean (SD)		13.8 (1.6)		14.6 (0.4)		15.6 (0.8)		12.6 (0.3)		
**Baseline scores, mean (SD)**										
	DASS depression	9.2 (10.4)	10.2 (9.6)	6.8 (7.1)	8.0 (8.0)	6.0 (3.0)	6.6 (3.2)	—	5.5 (6.6)	
	DASS anxiety	7.7 (6.9)	7.6 (5.8)	5.0 (5.6)	6.3 (5.8)	4.3 (4.2)	4.4 (2.6)	—	4.4 (2.2)	
	DASS stress	13.1 (8.6)	13.4 (6.1)	7.8 (5.9)	10.7 (8.0)	10.6 (4.0)	10.4 (4.3)	—	9.9 (6.2)	
	DASS total	30.2 (22.4)	31.1 (18.0)	19.7 (15.9)	25.0 (18.5)	20.9 (8.2)	21.3 (9.0)	—	19.4 (12.5)	
	Satisfaction (SLSS)	22.4 (6.4)	21.6 (6.2)	23.6 (4.8)	22.3 (6.0)	25.5 (2.3)	21.6 (6.7)	—	24.9 (4.6)	
	Flourishing (SWEMWBS)	17.4 (3.6)	16.0 (3.4)	19.1 (2.9)	17.7 (3.2)	19.0 (1.3)	17.6 (1.5)	—	17.0 (3.7)	

### Comparing Dropouts to Completers

Participants who dropped out were compared to completers on a series of independent-groups *t* tests. No significant differences were observed on the scales of depression (*P*=.19), anxiety (*P*=.66), stress (*P*=.85), DASS-total (*P*=.59), or the SWEMWBS (*P*=.05). A small difference (eta squared=0.01) was observed on the SLSS (*t*_321_=-2.0, *P*=.04), with dropouts reporting significantly less satisfaction (mean 21.8) than completers (mean 23.1).

The two schools that dropped out of the study provided reasons for ceasing to participate. The Anglican school B reported that the “overwhelming feedback from students had been negative” with the students finding both the control and Bite Back conditions “boring”. Students reported to their teachers that they did not seem to understand why they were doing this work, and more specifically, what benefits they would derive from their participation. They also reported that they found using the workbooks tedious and laborious. The Co-ed School ceased to participate in the study because the person in charge of implementing the study left 2 weeks after the study commenced and the other teachers did not continue with the research protocol.

### Adherence

Adherence was defined as the frequency with which students returned their completed workbook sections (ranging from 0-6): of 336 participants, 27 (8.0%) did not return any of their workbook sections, 52 participants (15.5%) returned 1-2 workbooks, 70 participants (20.8%) returned 3-4 workbooks, and 187 (55.6%) returned 5-6 workbooks.

Due to differences observed in the previous community Bite Back study on levels of engagement with the program, the current data were examined to determine if level of engagement affected outcomes. The sample was dichotomized according to the number of workbook sections completed. Participants who completed either 5 or 6 sections were considered “highly engaged”, while those who returned up to 4 completed sections of the workbook were categorized as “low engaged”. To ensure that this analysis was not affected by differential baseline scores, *t* tests were used to examine baseline differences on questionnaire scores between participants in the high versus low engagement groups. No significant differences between the groups were found.

### Universal Effects

#### Overview

To examine the effects of Bite Back on student well-being, an intent-to-treat analysis was completed using multilevel model analysis. Analyses of the universal effects were repeated with only the participants in the high engagement group.

#### Depression

Results indicated a non-significant interaction between time and condition for DASS-Depression scores: *F*_1,199.9_=.42, *P*=.52. The effect of time alone was significant, *F*_1,309.1_=8.10, *P*=.005, indicating a significant reduction of depression from baseline (mean 8.57) to post intervention (mean 6.93) for both conditions but no significant differences between the groups. Given that the baseline levels of psychopathology were low, the impact of the website on those reporting high levels of symptoms at the start of the program was also examined. The analysis was repeated among participants in the mild to extremely high categories of DASS depression baseline scores (n=226). No significant differences between the control and the Bite Back conditions were found. To examine if the level of engagement modified the results, the analysis was repeated to compare participants with low versus high engagement with the workbook sections. No significant differences were found between the groups.

#### Anxiety

There was no significant effect for DASS-Anxiety on the interaction of condition and time. The main effect for time was not significant. No significant interaction between condition and time was found among those with high baseline psychopathology scores, nor the highly engaged group.

#### Stress

The interaction between time and condition for DASS-Stress scores was not significant. However, the main effect for time was significant, *F*_1,188.4_=6.19, *P*=.01, suggesting a reduction in stress scores from baseline (mean 11.47) to post intervention (mean 10.37) for the entire sample. No significant time by condition interactions were observed among participants with high baseline scores. When the highly engaged participants were analyzed, a significant interaction of time and condition was observed: *F*_1,127.9_=5.56, *P*=.02. The highly engaged participants in the control group reported a reduction in stress from baseline (mean 12.44) to post intervention (mean 10.01), while the Bite Back participants reported a small increase from baseline (mean 9.49) to post intervention (mean 10.05).

#### DASS-Total

When total DASS scores were examined, the interaction between time and condition was not significant. However, there was a significant effect of time across both groups, *F*_1,184.57_=6.67, *P*=.01, with reductions in total DASS scores from baseline (mean 26.69) to post intervention (mean 23.65) for both conditions. When participants with high baseline scores were analyzed alone, this interaction was not significant: *F*_1,57.75_=.29, *P*=.60. For participants who were highly engaged, the interaction of condition and time was not significant: *F*_1,126.47_=3.11, *P*=.08.

#### Life Satisfaction

The interaction of time and condition for the SLSS was not significant. However, the main effect of time was significant, *F*_1,247.0_=17.35, *P*<.001, with participants reporting a reduction of life satisfaction from baseline (mean 22.56) to post intervention (mean 21.58). When highly engaged participants were analyzed, the time by condition interaction was significant: *F*_1,153.22_=6.57, *P*=.01. Highly engaged control participants reported little change in life satisfaction (mean 21.9 at baseline compared to mean 22.09 at post intervention), while the Bite Back participants reported a larger reduction from baseline (mean 23.99) to post intervention (mean 21.90).

#### Flourishing

The interaction between time and condition on the SWEMWBS was significant: *F*_1,202.9_=5.88, *P*=.02. Baseline scores of participants in the control condition (mean 16.68) increased at post intervention (mean 18.19), while the Bite Back group reported little change (mean 18.18 at baseline increasing to mean 18.37 at post intervention). A significant difference in the interaction of time and condition was also observed when the group with high engagement was analyzed separately: *F*_1,150.4_=4.15, *P*=.04.

## Discussion

### Principal Findings

The aim of the current study was to evaluate the feasibility and effectiveness of the Bite Back program when delivered to adolescents in a classroom environment. Results indicated that Bite Back did not lead to any significant improvement in mental health outcomes compared to the control condition. For the flourishing scale, there was a significant difference between the control group and Bite Back across the two time points, favoring control. However, the control condition started the intervention with lower scores and returned to the same level as the Bite Back condition post intervention, so it is difficult to establish if this was a case of regression to the mean or if there was a more positive impact of the control condition compared to Bite Back. The principle conclusion that can be drawn from these results is that there was no significant benefit of participating in the Bite Back condition compared to the control condition.

Previous findings from this team in a community sample of young people suggests that when Bite Back is delivered in an unstructured format and with freedom to choose engagement frequency, it led to reductions in mental health symptoms and improvements in well-being compared to the control condition [9,10]. The sample’s age was comparable to our study sample as well as the baseline levels of psychopathology and well-being. However, our findings indicate that when the same program is delivered in a structured method and participants are obliged to use the website at a prescribed frequency, there is no benefit from the Bite Back. A plausible explanation for the current findings is that it is the conversion of Bite Back into a structured school program that explains the observed results.

There are several aspects of the conversion that may have contributed to this. In the current study, participants were required to use Bite Back several times each week. Some students may have resented the pressure to interact with the program on a regular basis. Previous research has found that the benefits of acts of kindness and expressing gratitude disappeared when participants were asked to complete them more than once per week [30]. The authors of this study suggested that dose may be important when the participants were not free to choose the specific activity [31]. Other research has found that in an online positive psychology intervention completing 2-4 exercises improved well-being but attempting to complete up to 6 exercises negated all benefits [32]. These authors concluded that participants should not be overwhelmed with activities. Our findings suggest that in addition to these factors, being forced to engage in positive psychology may also remove its beneficial effects.

It has been previously found that individual factors such as motivation and effortful engagement are important in order to obtain benefits from positive psychology [33-35]. Level of engagement and choice of activity in the community study were up to participants, whereas in the current study, students were required to complete the exercises as part of their curricular activities at school. The greater level of motivation that can be expected when participants are completely free to choose their level of engagement in the program may have played a role in our findings.

Finally, the differential usage of specific aspects of Bite Back may have affected these findings. While the Bite Back program involves several activities relating to numerous positive psychology domains, there have been mixed findings in relation to the effectiveness of domain-specific well-being programs. Mindfulness, for example, has a wealth of research supporting its effectiveness as an intervention strategy when used in isolation [36,37]. However, the domain of optimism when used in isolation has significantly fewer supporting studies to date. Participants in the community study with free access to Bite Back content may have engaged to a greater extent with more “active ingredients”, such as mindfulness or with activities that piqued their interest, while participants in this study were required to engage with each positive psychology domain regardless of their interest. This may have dampened the treatment effects for the current study.

Our study highlights that the method of delivery of youth mental health programs may be equally important as content. Future research would also be warranted to clarify which delivery-specific factors may have been implicated in our study. While a number of such factors were considered in relation to these findings, only future research could determine whether these were indeed implicated and to what extent. It is possible that there were other delivery-specific factors not considered by us. Such research would benefit other school programs, which could then be tailored to accommodate these influences and also help transition programs from open-access to becoming more structured and school-based. In addition, while it appears plausible that delivery-specific factors account for our findings, it cannot be excluded that this study is a true reflection of the efficacy of Bite Back. Future trials to clarify this question would also be beneficial.

### Limitations

The findings of this study should be interpreted in light of its limitations. The sample used in this study was a relatively heterogeneous sample with participants coming from schools that were both co-ed and single gender and that reflected different religious denominations and philosophies. There were relatively few checks on how the program was presented across the various schools and classrooms. This could be both a strength and limitation of the study, as it more closely replicated real-life applications of positive psychology in the school environment.

The sample size was relatively small, although this is usual in feasibility type research as it provides data for further large-scale studies. The study was also limited by a lack of long-term follow-up data, although it is common for the strongest reduction in symptoms to be observed post intervention and so it is likely that significant differences would not have appeared at a later time point.

### Conclusion

The application of the online positive psychology program, Bite Back, into a structured classroom intervention did not demonstrate significant benefits when compared to a control intervention. This may not be a true reflection of the efficacy of the program but rather a problem in the application of Bite Back to the school environment.

## Appendix

Multimedia Appendix 1 CONSORT-EHEALTH checklist V1.6.2 [17].

## Abbreviations

DASS-21: Depression, Anxiety, and Stress Scale—Short Form
SWEMWBS: Short Warwick Edinburgh Mental Well-Being Scale
SLSS: Student Life Satisfaction Scale
