# Supplementary material for: A Web-Based Adolescent Positive Psychology Program in Schools: Randomized Controlled Trial
Source: J Med Internet Res. 2015 Jul 28;17(7):e187. doi: 10.2196/jmir.4329 (PMC4705364; doi:10.2196/jmir.4329)
Supplement: Multimedia Appendix 1 [file jmir_v17i7e187_app1.pdf]

CONSORT-EHEALTH (V 1.6.1) - Submission/Publication Form

The CONSORT-EHEALTH checklist is intended for authors of randomized trials evaluating web-based and Internet-based applications/interventions, including mobile interventions, electronic games (incl multiplayer games), social media, certain telehealth applications, and other interactive and/or networked electronic applications. Some of the items (e.g. all subitems under item 5 - description of the intervention) may also be applicable for other study designs.

The goal of the CONSORT EHEALTH checklist and guideline is to be
a) a guide for reporting for authors of RCTs,
b) to form a basis for appraisal of an ehealth trial (in terms of validity)

CONSORT-EHEALTH items/subitems are MANDATORY reporting items for studies published in the Journal of Medical Internet Research and other journals / scientific societies endorsing the checklist.

Items numbered 1., 2., 3., 4a., 4b etc are original CONSORT or CONSORT-NPT (non-pharmacologic treatment) items.
Items with Roman numerals (i., ii, iii, iv etc.) are CONSORT-EHEALTH extensions/clarifications.

As the CONSORT-EHEALTH checklist is still considered in a formative stage, we would ask that you also RATE ON A SCALE OF 1-5 how important/useful you feel each item is FOR THE PURPOSE OF THE CHECKLIST and reporting guideline (optional).

Mandatory reporting items are marked with a red *.
In the textboxes, either copy & paste the relevant sections from your manuscript into this form - please include any quotes from your manuscript in QUOTATION MARKS,
or answer directly by providing additional information not in the manuscript, or elaborating on why the item was not relevant for this study.

YOUR ANSWERS WILL BE PUBLISHED AS A SUPPLEMENTARY FILE TO YOUR PUBLICATION IN JMIR AND ARE CONSIDERED PART OF YOUR PUBLICATION (IF ACCEPTED).
Please fill in these questions diligently. Information will not be copyedited, so please use proper spelling and grammar, use correct capitalization, and avoid abbreviations.

DO NOT FORGET TO SAVE AS PDF _AND_ CLICK THE SUBMIT BUTTON SO YOUR ANSWERS ARE IN OUR DATABASE !!!

Citation Suggestion (if you append the pdf as Appendix we suggest to cite this paper in the caption):
Eysenbach G, CONSORT-EHEALTH Group
CONSORT-EHEALTH: Improving and Standardizing Evaluation Reports of Web-based and Mobile Health Interventions
J Med Internet Res 2011;13(4):e126
URL: http://www.jmir.org/2011/4/e126/
doi: 10.2196/jmir.1923
PMID: 22209829

* Required

Your name *

First Last

Burckhardt

Primary Affiliation (short), City, Country *

University of Toronto, Toronto, Canada

University of NSW and

Your e-mail address *

abc@gmail.com

r.burckhardt@unsw.ed

Title of your manuscript *

Provide the (draft) title of your manuscript.

Feasibility of an Adolescent Web-Based Positive Psychology Program in schools: A Randomized Controlled Trial

Article Preparation Status/Stage *

At which stage in your article preparation are you currently (at the time you fill in this form)

- ( ) not submitted yet - in early draft status
- (•) not submitted yet - in late draft status, just before submission
- ( ) submitted to a journal but not reviewed yet
- ( ) submitted to a journal and after receiving initial reviewer comments
- ( ) submitted to a journal and accepted, but not published yet
- ( ) published
- ( ) Other:

Journal *

If you already know where you will submit this paper (or if it is already submitted), please provide the journal name (if it is not JMIR, provide the journal name under "other")

- ( ) not submitted yet / unclear where I will submit this
- (•) Journal of Medical Internet Research (JMIR)
- ( ) Other:

Manuscript tracking number *

If this is a JMIR submission, please provide the manuscript tracking number under "other" (The ms tracking number can be found in the submission acknowledgement email, or when you login as author in JMIR. If the paper is already published in JMIR, then the ms tracking number is the four-digit number at the end of the DOI, to be found at the bottom of each published article in JMIR)

- (•) no ms number (yet) / not (yet) submitted to / published in JMIR
- ( ) Other:

TITLE AND ABSTRACT

1a) TITLE: Identification as a randomized trial in the title

1a) Does your paper address CONSORT item 1a? *

I.e does the title contain the phrase "Randomized Controlled Trial"? (if not, explain the reason under "other")

- (•) yes
- ( ) Other:

1a-i) Identify the mode of delivery in the title

Identify the mode of delivery. Preferably use "web-based" and/or "mobile" and/or "electronic game" in the title. Avoid ambiguous terms like "online", "virtual", "interactive". Use "Internet-based" only if Intervention includes non-web-based Internet components (e.g. email), use "computer-based" or "electronic" only if offline products are used. Use "virtual" only in the context of "virtual reality" (3-D worlds). Use "online" only in the context of "online support groups". Complement or substitute product names with broader terms for the class of products (such as "mobile" or "smart phone" instead of "iphone"), especially if the application runs on different platforms.

	1	2	3	4	5	
subitem not at all important	( )	( )	( )	( )	( )	essential

Does your paper address subitem 1a-i? *

Copy and paste relevant sections from manuscript title (include quotes in quotation marks "like this" to indicate direct quotes from your manuscript), or elaborate on this item by providing additional information not in the ms, or briefly explain why the item is not applicable/relevant for your study

Yes it uses the words "web-based" in the title

1a-ii) Non-web-based components or important co-interventions in title

Mention non-web-based components or important co-interventions in title, if any (e.g., "with telephone support").

	1	2	3	4	5	
subitem not at all important	( )	( )	( )	( )	( )	essential

Does your paper address subitem 1a-ii?

Copy and paste relevant sections from manuscript title (include quotes in quotation marks "like this" to indicate direct quotes from your manuscript), or elaborate on this item by providing additional information not in the ms, or briefly explain why the item is not applicable/relevant for your study

1a-iii) Primary condition or target group in the title

Mention primary condition or target group in the title, if any (e.g., “for children with Type I Diabetes”) Example: A Web-based and Mobile Intervention with Telephone Support for Children with Type I Diabetes: Randomized Controlled Trial

	1	2	3	4	5	
subitem not at all important	○	○	○	○	○	essential

Does your paper address subitem 1a-iii? *

Copy and paste relevant sections from manuscript title (include quotes in quotation marks "like this" to indicate direct quotes from your manuscript), or elaborate on this item by providing additional information not in the ms, or briefly explain why the item is not applicable/relevant for your study

Yes. It uses the words "adolescent" and "schools" in the title

1b) ABSTRACT: Structured summary of trial design, methods, results, and conclusions

NPT extension: Description of experimental treatment, comparator, care providers, centers, and blinding status.

1b-i) Key features/functionalities/components of the intervention and comparator in the METHODS section of the ABSTRACT

Mention key features/functionalities/components of the intervention and comparator in the abstract. If possible, also mention theories and principles used for designing the site. Keep in mind the needs of systematic reviewers and indexers by including important synonyms. (Note: Only report in the abstract what the main paper is reporting. If this information is missing from the main body of text, consider adding it)

	1	2	3	4	5	
subitem not at all important	○	○	○	○	○	essential

Does your paper address subitem 1b-i? *

Copy and paste relevant sections from the manuscript abstract (include quotes in quotation marks "like this" to indicate direct quotes from your manuscript), or elaborate on this item by providing additional information not in the ms, or briefly explain why the item is not applicable/relevant for your study

Yes. The following is stated "Students from four high schools were randomly allocated by classroom to either the positive psychology condition, 'Bite Back', or the control condition. The Bite Back condition comprised of positive psychology exercises and information while the control condition utilized a series of non-psychology entertainment websites. Both interventions were delivered online for six hours over a four- to six-week period during class time. Symptom measures and measures of wellbeing, life satisfaction and flourishing were administered at baseline and at 6-week post-intervention."

1b-ii) Level of human involvement in the METHODS section of the ABSTRACT

Clarify the level of human involvement in the abstract, e.g., use phrases like "fully automated" vs. "therapist/nurse/care provider/physician-assisted" (mention number and expertise of providers involved, if any). (Note: Only report in the abstract what the main paper is reporting. If this information is missing from the main body of text, consider adding it)

	1	2	3	4	5	
subitem not at all important	◯	◯	◯	◯	◯	essential

Does your paper address subitem 1b-ii?

Copy and paste relevant sections from the manuscript abstract (include quotes in quotation marks "like this" to indicate direct quotes from your manuscript), or elaborate on this item by providing additional information not in the ms, or briefly explain why the item is not applicable/relevant for your study

1b-iii) Open vs. closed, web-based (self-assessment) vs. face-to-face assessments in the METHODS section of the ABSTRACT

Mention how participants were recruited (online vs. offline), e.g., from an open access website or from a clinic or a closed online user group (closed usergroup trial), and clarify if this was a purely web-based trial, or there were face-to-face components (as part of the intervention or for assessment). Clearly say if outcomes were self-assessed through questionnaires (as common in web-based trials). Note: In traditional offline trials, an open trial (open-label trial) is a type of clinical trial in which both the researchers and participants know which treatment is being administered. To avoid confusion, use "blinded" or "unblinded" to indicated the level of blinding instead of "open", as "open" in web-based trials usually refers to "open access" (i.e. participants can self-enrol). (Note: Only report in the abstract what the main paper is reporting. If this information is missing from the main body of text, consider adding it)

	1	2	3	4	5	
subitem not at all important	◯	◯	◯	◯	◯	essential

Does your paper address subitem 1b-iii?

Copy and paste relevant sections from the manuscript abstract (include quotes in quotation marks "like this" to indicate direct quotes from your manuscript), or elaborate on this item by providing

additional information not in the ms, or briefly explain why the item is not applicable/relevant for your study

1b-iv) RESULTS section in abstract must contain use data

Report number of participants enrolled/assessed in each group, the use/uptake of the intervention (e.g., attrition/adherence metrics, use over time, number of logins etc.), in addition to primary/secondary outcomes. (Note: Only report in the abstract what the main paper is reporting. If this information is missing from the main body of text, consider adding it)

	1	2	3	4	5	
subitem not at all important	◯	◯	◯	◯	◯	essential

Does your paper address subitem 1b-iv?

Copy and paste relevant sections from the manuscript abstract (include quotes in quotation marks "like this" to indicate direct quotes from your manuscript), or elaborate on this item by providing additional information not in the ms, or briefly explain why the item is not applicable/relevant for your study

1b-v) CONCLUSIONS/DISCUSSION in abstract for negative trials

Conclusions/Discussions in abstract for negative trials: Discuss the primary outcome - if the trial is negative (primary outcome not changed), and the intervention was not used, discuss whether negative results are attributable to lack of uptake and discuss reasons. (Note: Only report in the abstract what the main paper is reporting. If this information is missing from the main body of text, consider adding it)

	1	2	3	4	5	
subitem not at all important	◯	◯	◯	◯	◯	essential

Does your paper address subitem 1b-v?

Copy and paste relevant sections from the manuscript abstract (include quotes in quotation marks "like this" to indicate direct quotes from your manuscript), or elaborate on this item by providing additional information not in the ms, or briefly explain why the item is not applicable/relevant for your study

INTRODUCTION

2a) In INTRODUCTION: Scientific background and explanation of rationale

2a-i) Problem and the type of system/solution

Describe the problem and the type of system/solution that is object of the study: intended as stand-alone intervention vs. incorporated in broader health care program? Intended for a particular patient population? Goals of the intervention, e.g., being more cost-effective to other interventions, replace or complement other solutions? (Note: Details about the intervention are provided in “Methods” under 5)

	1	2	3	4	5	
subitem not at all important	○	○	○	○	○	essential

Does your paper address subitem 2a-i? *

Copy and paste relevant sections from the manuscript (include quotes in quotation marks "like this" to indicate direct quotes from your manuscript), or elaborate on this item by providing additional information not in the ms, or briefly explain why the item is not applicable/relevant for your study

Yes. The following are included which describe the problem:

"Epidemiological data indicates that in Australia one in four young people between the ages of 16 to 24 years have experienced at least one mental disorder in the preceding year and that young people have the highest rates of mental disorders compared to any other age group [1]. It has been estimated that of those aged 16 to 24 years who experiencing mental health issues, 77% did not access any medical or professional services in the preceding 12 months and that they account for almost 30% of the total mental health burden in Australia [2, 3]. This is further aggravated by their relative lack of skills in dealing with life stresses and emotional distress compared to adults [4].

A number of early intervention programs have been developed to address skills deficits and to build resilience in young people. Most of these programs have utilized cognitive-behavioral therapy techniques with varying degress of success [5]. This suggests that researchers need to look further afield for effective prevention approaches to youth mental health such as the building of skills and strategies to enhance personal strengths [6]."

2a-ii) Scientific background, rationale: What is known about the (type of) system

Scientific background, rationale: What is known about the (type of) system that is the object of the study (be sure to discuss the use of similar systems for other conditions/diagnoses, if appropiate), motivation for the study, i.e. what are the reasons for and what is the context for this specific study, from which stakeholder viewpoint is the study performed, potential impact of findings [2]. Briefly justify the choice of the comparator.

	1	2	3	4	5	
subitem not at all important	○	○	○	○	○	essential

Does your paper address subitem 2a-ii? *

Copy and paste relevant sections from the manuscript (include quotes in quotation marks "like this" to indicate direct quotes from your manuscript), or elaborate on this item by providing additional information not in the ms, or briefly explain why the item is not applicable/relevant for your study

Yes, there is information that outlines the current state of knowledge about the particular 'system' which in this case is positive psychology. See quote below:

"In this regard, positive psychology, the scientific study of happiness, wellbeing, and flourishing, may offer an alternative to conventional prevention programs for youth. Positive psychology can be conceptualized as a range of behavioral, attitudinal and emotional domains that are important for improving wellbeing. These include: developing healthy social relationships, becoming more optimism, finding ‘meaning’ and practicing mindfulness. Evaluations of non-school-based programs have reported on the benefits of engaging in positive psychology exercises. For example, interventions that increase hope have been shown to predict lower illicit substance use, lower levels of depression, anxiety and hostility, less behavioral problems, and higher academic performance in adolescents [7, 8].

Manicavasagar et al., conducted a feasibility study of a community-based online positive psychological program ‘Bite Back’ for adolescents aged between 12-18 year [9, 10]. Bite Back comprised information and interactive activities relating to nine domains: gratitude, optimism, flow, meaning, hope, mindfulness, character strengths, healthy lifestyle, and positive relationships. Participants in both the Bite Back condition and a control condition were encouraged to regularly use their respective websites over a six-week period. Symptom measures of depression, anxiety and stress, and wellbeing were compared within and between conditions and at baseline and post-intervention. At the start of the intervention, participants reported low levels of psychopathology, as was expected in a non-clinical sample. Results demonstrated significant decreases in symptom scores and increased scores on wellbeing post-intervention for the Bite Back relative to the control condition. It was reported that when participants with high levels of engagement were examined in isolation, the findings were even stronger suggesting that increased usage was related to greater benefits. Furthermore, qualitative analyses indicated that Bite Back was well received by participants allocated to this condition. It is notable that participants using this online positive psychology program could freely engage with the material at their own pace and in their own time. However, the open-access online format of this program meant that the researchers were unable to examine how participants navigated the website and whether they were exposed to the full range of material and information about positive psychology.

A literature search did not uncover any previously evaluated computer-based school-delivered positive psychology interventions. However group format school-based positive psychology interventions, such as the Positive Psychology Program, Wellness-Promotion Intervention and others, consist of lesson-based modules pertaining to specific topics and skills delivered over the intervention period [11-16]. It was therefore deemed timely to evaluate Bite Back in a structured lesson-based format that was appropriate for delivery within a school environment. This study explores the feasibility of implementing a structured workbook-guided version of Bite Back in senior schools as part of the schools’ curriculum. It was expected that the benefits found in our previous study would be extended to this student population, specifically, that participants would improve on measures of wellbeing and report decreased symptoms scores following the intervention compared to a control condition."

2b) In INTRODUCTION: Specific objectives or

hypotheses

Does your paper address CONSORT subitem 2b? *

Copy and paste relevant sections from the manuscript (include quotes in quotation marks "like this" to indicate direct quotes from your manuscript), or elaborate on this item by providing additional information not in the ms, or briefly explain why the item is not applicable/relevant for your study

Yes, the introduction contains specific objectives/hypothesis. See below:

"It was therefore deemed timely to evaluate Bite Back in a structured lesson-based format that was appropriate for delivery within a school environment. This study explores the feasibility of implementing a structured workbook-guided version of Bite Back in senior schools as part of the schools' curriculum. It was expected that the benefits found in our previous study would be extended to this student population, specifically, that participants would improve on measures of wellbeing and report decreased symptoms scores following the intervention compared to a control condition."

METHODS

3a) Description of trial design (such as parallel, factorial) including allocation ratio

Does your paper address CONSORT subitem 3a? *

Copy and paste relevant sections from the manuscript (include quotes in quotation marks "like this" to indicate direct quotes from your manuscript), or elaborate on this item by providing additional information not in the ms, or briefly explain why the item is not applicable/relevant for your study

Yes. See quotes below:

"Between the dates of January 2012 and March 2012, 20 schools were invited to participate in this study, of which four agreed. Prior to study commencement, the lead author met with senior school staff and welfare officers to convey the requirements of the study and to instruct teachers in the study methodology. Parental and student self-consent were obtained for all participants under the age of 16 years and student self-consent only was obtained for participants aged 16 years and over. The year group designated to participate in the study was chosen by the school and no exclusion criteria were applied.

Teachers were instructed to explain to students that they were participating in a study designed to examine the way in which websites affect how young people view the world. Teachers were also required to provide a 5 minute discussion on this topic prior to the beginning of the study and were asked not to mention that the study included a positive psychology intervention. Teachers were responsible for handing out workbooks, providing students with access to relevant websites, and managing students' behavior during class time. Teachers were unaware as to which classes had been allocated to the intervention or control conditions. Students had no face-to-face contact with any of the researchers prior to, during, or following the study.

Students were randomized by blocks (classes) using an Excel random number generator to allocate them to either the Bite Back or control conditions by an independent researcher not associated with the data collection. Equivalent numbers of students were allocated to each condition across each year group and for each school (e.g. Year 9 at the Boys School). In cases where there were uneven numbers of blocks, students were assigned to the Bite Back condition. Students completed an online battery of self-report questionnaires twice during class time: pre- and post-intervention.

All schools were instructed to use the workbook over a six-week period. However, two schools (the Boys School and the Co-ed School) completed the workbook over a four week period due to class-time constraints. Teachers were told that a total of six hours of face-to-face time was required from students over a period of four to six weeks. After each session, students in both the control and Bite Back conditions were asked to email completed sections of their workbooks to the researchers. This strategy was employed to encourage engagement and increase compliance. It also served as an indicator of student adherence to the research tasks.

3b) Important changes to methods after trial commencement (such as eligibility criteria), with reasons

Does your paper address CONSORT subitem 3b? *

Copy and paste relevant sections from the manuscript (include quotes in quotation marks "like this" to indicate direct quotes from your manuscript), or elaborate on this item by providing additional information not in the ms, or briefly explain why the item is not applicable/relevant for your study

There were no changes to methods after trial commencement

3b-i) Bug fixes, Downtimes, Content Changes

Bug fixes, Downtimes, Content Changes: ehealth systems are often dynamic systems. A description of changes to methods therefore also includes important changes made on the intervention or comparator during the trial (e.g., major bug fixes or changes in the functionality or content) (5-iii) and other “unexpected events” that may have influenced study design such as staff changes, system failures/downtimes, etc. [2].

	1	2	3	4	5	
subitem not at all important	○	○	○	○	○	essential

Does your paper address subitem 3b-i?

Copy and paste relevant sections from the manuscript (include quotes in quotation marks "like this" to indicate direct quotes from your manuscript), or elaborate on this item by providing additional information not in the ms, or briefly explain why the item is not applicable/relevant for your study

4a) Eligibility criteria for participants

Does your paper address CONSORT subitem 4a? *

Copy and paste relevant sections from the manuscript (include quotes in quotation marks "like this" to indicate direct quotes from your manuscript), or elaborate on this item by providing additional information not in the ms, or briefly explain why the item is not applicable/relevant for your study

Yes. See below:

"Four Australian high schools - two Anglican girls schools (referred to as Girls School A and Girls School B), a Catholic boys school (referred to as Boys School), and a Jewish co-educational school (referred to as Co-ed School) agreed to participate in the study. Students were in Years 7 through to 12 which in the Australian educative system are the final 6 years before being eligible to go to university. These four schools were amongst the highest in terms of socioeconomic status compared to other schools in Australia. Students were included in the study only if they completed the relevant consent forms. This study was approved by the UNSW Human Research Ethics Advisory Committee (Ethics Approval number 2011-7-35)."

4a-i) Computer / Internet literacy

Computer / Internet literacy is often an implicit “de facto” eligibility criterion - this should be explicitly clarified.

	1	2	3	4	5	
subitem not at all important	○	○	○	○	○	essential

Does your paper address subitem 4a-i?

Copy and paste relevant sections from the manuscript (include quotes in quotation marks "like this" to indicate direct quotes from your manuscript), or elaborate on this item by providing additional information not in the ms, or briefly explain why the item is not applicable/relevant for your study

4a-ii) Open vs. closed, web-based vs. face-to-face assessments:

Open vs. closed, web-based vs. face-to-face assessments: Mention how participants were recruited (online vs. offline), e.g., from an open access website or from a clinic, and clarify if this was a purely web-based trial, or there were face-to-face components (as part of the intervention or for assessment), i.e., to what degree got the study team to know the participant. In online-only trials, clarify if participants were quasi-anonymous and whether having multiple identities was possible or whether technical or logistical measures (e.g., cookies, email confirmation, phone calls) were used to detect/prevent these.

	1	2	3	4	5	
subitem not at all important	○	○	○	○	○	essential

Does your paper address subitem 4a-ii? *

Copy and paste relevant sections from the manuscript (include quotes in quotation marks "like this" to indicate direct quotes from your manuscript), or elaborate on this item by providing additional information not in the ms, or briefly explain why the item is not applicable/relevant for your study

A description of how participants were recruited was included:

"Four Australian high schools - two Anglican girls schools (referred to as Girls School A and Girls School B), a Catholic boys school (referred to as Boys School), and a Jewish co-educational school (referred to as Co-ed School) agreed to participate in the study. Students were in Years 7 through to 12 which in the Australian educative system are the final 6 years before being eligible to go to university. These four schools were amongst the highest in terms of socioeconomic status compared to other schools in Australia. Students were included in the study only if they completed the relevant consent forms. This study was approved by the UNSW Human Research Ethics Advisory Committee (Ethics Approval number 2011-7-35)."

"Between the dates of January 2012 and March 2012, 20 schools were invited to participate in this study, of which four agreed. Prior to study commencement, the lead author met with senior school staff and welfare officers to convey the requirements of the study and to instruct teachers in the study methodology. Parental and student self-consent were obtained for all participants under the age of 16 years and student self-consent only was obtained for participants aged 16 years and over. The year group designated to participate in the study was chosen by the school and no exclusion criteria were applied.

Teachers were instructed to explain to students that they were participating in a study designed to examine the way in which websites affect how young people view the world. Teachers were also required to provide a 5 minute discussion on this topic prior to the beginning of the study and were asked not to mention that the study included a positive psychology intervention. Teachers were responsible for handing out workbooks, providing students with access to relevant websites, and managing students' behavior during class time. Teachers were unaware as to which classes had been allocated to the intervention or control conditions. Students had no face-to-face contact with any of the researchers prior to, during, or following the study.

Students were randomized by blocks (classes) using an Excel random number generator to allocate them to either the Bite Back or control conditions by an independent researcher not associated with the data collection. Equivalent numbers of students were allocated to each condition across each year group and for each school (e.g. Year 9 at the Boys School). In cases where there were uneven numbers of blocks, students were assigned to the Bite Back condition. Students completed an online battery of self-report questionnaires twice during class time: pre- and post-intervention.

All schools were instructed to use the workbook over a six-week period. However, two schools (the Boys School and the Co-ed School) completed the workbook over a four week period due to class-time constraints. Teachers were told that a total of six hours of face-to-face time was required from students over a period of four to six weeks. After each session, students in both the control and Bite Back conditions were asked to email completed sections of their workbooks to the researchers. This strategy was employed to encourage engagement and increase compliance. It also served as an indicator of student adherence to the research tasks.

4a-iii) Information giving during recruitment

Information given during recruitment. Specify how participants were briefed for recruitment and in the informed consent procedures (e.g., publish the informed consent documentation as appendix, see also item X26), as this information may have an effect on user self-selection, user expectation and may also bias results.

	1	2	3	4	5	
subitem not at all important	○	○	○	○	○	essential

Does your paper address subitem 4a-iii?

Copy and paste relevant sections from the manuscript (include quotes in quotation marks "like this" to indicate direct quotes from your manuscript), or elaborate on this item by providing additional information not in the ms, or briefly explain why the item is not applicable/relevant for your study

4b) Settings and locations where the data were collected

Does your paper address CONSORT subitem 4b? *

Copy and paste relevant sections from the manuscript (include quotes in quotation marks "like this" to indicate direct quotes from your manuscript), or elaborate on this item by providing additional information not in the ms, or briefly explain why the item is not applicable/relevant for your study

A description of the settings and location of data collection was included:

"Four Australian high schools - two Anglican girls schools (referred to as Girls School A and Girls School B), a Catholic boys school (referred to as Boys School), and a Jewish co-educational school (referred to as Co-ed School) agreed to participate in the study. Students were in Years 7 through to 12 which in the Australian educative system are the final 6 years before being eligible to go to university. These four schools were amongst the highest in terms of socioeconomic status compared to other schools in Australia. Students were included in the study only if they completed the relevant consent forms. This study was approved by the UNSW Human Research Ethics Advisory Committee (Ethics Approval number 2011-7-35)."

"Between the dates of January 2012 and March 2012, 20 schools were invited to participate in this study, of which four agreed. Prior to study commencement, the lead author met with senior school staff and welfare officers to convey the requirements of the study and to instruct teachers in the study methodology. Parental and student self-consent were obtained for all participants under the age of 16 years and student self-consent only was obtained for participants aged 16 years and over. The year group designated to participate in the study was chosen by the school and no exclusion criteria were applied.

Teachers were instructed to explain to students that they were participating in a study designed to examine the way in which websites affect how young people view the world. Teachers were also required to provide a 5 minute discussion on this topic prior to the beginning of the study and were asked not to mention that the study included a positive psychology intervention. Teachers were responsible for handing out workbooks, providing students with access to relevant websites, and managing students' behavior during class time. Teachers were unaware as to which classes had been allocated to the intervention or control conditions. Students had no face-to-face contact with any of the researchers prior to, during, or following the study.

Students were randomized by blocks (classes) using an Excel random number generator to allocate them to either the Bite Back or control conditions by an independent researcher not associated with the data collection. Equivalent numbers of students were allocated to each condition across each year group and for each school (e.g. Year 9 at the Boys School). In cases where there were uneven numbers of blocks, students were assigned to the Bite Back condition. Students completed an online battery of self-report questionnaires twice during class time: pre- and post-intervention.

All schools were instructed to use the workbook over a six-week period. However, two schools (the Boys School and the Co-ed School) completed the workbook over a four week period due to class-time constraints. Teachers were told that a total of six hours of face-to-face time was required from students over a period of four to six weeks. After each session, students in both the control and Bite Back conditions were asked to email completed sections of their workbooks to the researchers. This strategy was employed

4b-i) Report if outcomes were (self-)assessed through online questionnaires

Clearly report if outcomes were (self-)assessed through online questionnaires (as common in web-based trials) or otherwise.

1 2 3 4 5

subitem not at all important ◯ ◯ ◯ ◯ ◯ essential

Does your paper address subitem 4b-i? *

Copy and paste relevant sections from the manuscript (include quotes in quotation marks "like this" to indicate direct quotes from your manuscript), or elaborate on this item by providing additional information not in the ms, or briefly explain why the item is not applicable/relevant for your study

Yes, it was online and it was reported in the manuscript: "Students completed an online battery of self-report questionnaires twice during class time: pre- and post-intervention."

4b-ii) Report how institutional affiliations are displayed

Report how institutional affiliations are displayed to potential participants [on ehealth media], as affiliations with prestigious hospitals or universities may affect volunteer rates, use, and reactions with regards to an intervention.(Not a required item – describe only if this may bias results)

1 2 3 4 5

subitem not at all important ◯ ◯ ◯ ◯ ◯ essential

Does your paper address subitem 4b-ii?

Copy and paste relevant sections from the manuscript (include quotes in quotation marks "like this" to indicate direct quotes from your manuscript), or elaborate on this item by providing additional information not in the ms, or briefly explain why the item is not applicable/relevant for your study

5) The interventions for each group with sufficient details to allow replication, including how and when they were actually administered

5-i) Mention names, credential, affiliations of the developers, sponsors, and owners

Mention names, credential, affiliations of the developers, sponsors, and owners [6] (if authors/evaluators are owners or developer of the software, this needs to be declared in a “Conflict of interest” section or mentioned elsewhere in the manuscript).

1 2 3 4 5

subitem not at all important ◯ ◯ ◯ ◯ ◯ essential

Does your paper address subitem 5-i?

Copy and paste relevant sections from the manuscript (include quotes in quotation marks "like this" to indicate direct quotes from your manuscript), or elaborate on this item by providing additional information not in the ms, or briefly explain why the item is not applicable/relevant for your study

5-ii) Describe the history/development process

Describe the history/development process of the application and previous formative evaluations (e.g., focus groups, usability testing), as these will have an impact on adoption/use rates and help with interpreting results.

	1	2	3	4	5	
subitem not at all important	◯	◯	◯	◯	◯	essential

Does your paper address subitem 5-ii?

Copy and paste relevant sections from the manuscript (include quotes in quotation marks "like this" to indicate direct quotes from your manuscript), or elaborate on this item by providing additional information not in the ms, or briefly explain why the item is not applicable/relevant for your study

5-iii) Revisions and updating

Revisions and updating. Clearly mention the date and/or version number of the application/intervention (and comparator, if applicable) evaluated, or describe whether the intervention underwent major changes during the evaluation process, or whether the development and/or content was "frozen" during the trial. Describe dynamic components such as news feeds or changing content which may have an impact on the replicability of the intervention (for unexpected events see item 3b).

	1	2	3	4	5	
subitem not at all important	◯	◯	◯	◯	◯	essential

Does your paper address subitem 5-iii?

Copy and paste relevant sections from the manuscript (include quotes in quotation marks "like this" to indicate direct quotes from your manuscript), or elaborate on this item by providing additional information not in the ms, or briefly explain why the item is not applicable/relevant for your study

5-iv) Quality assurance methods

Provide information on quality assurance methods to ensure accuracy and quality of information provided [1], if applicable.

	1	2	3	4	5	
subitem not at all important	◯	◯	◯	◯	◯	essential

Does your paper address subitem 5-iv?

Copy and paste relevant sections from the manuscript (include quotes in quotation marks "like this" to indicate direct quotes from your manuscript), or elaborate on this item by providing additional information not in the ms, or briefly explain why the item is not applicable/relevant for your study

5-v) Ensure replicability by publishing the source code, and/or providing screenshots/screen-capture video, and/or providing flowcharts of the algorithms used

Ensure replicability by publishing the source code, and/or providing screenshots/screen-capture video, and/or providing flowcharts of the algorithms used. Replicability (i.e., other researchers should in principle be able to replicate the study) is a hallmark of scientific reporting.

	1	2	3	4	5	
subitem not at all important	◯	◯	◯	◯	◯	essential

Does your paper address subitem 5-v?

Copy and paste relevant sections from the manuscript (include quotes in quotation marks "like this" to indicate direct quotes from your manuscript), or elaborate on this item by providing additional information not in the ms, or briefly explain why the item is not applicable/relevant for your study

5-vi) Digital preservation

Digital preservation: Provide the URL of the application, but as the intervention is likely to change or

disappear over the course of the years; also make sure the intervention is archived (Internet Archive, webcitation.org, and/or publishing the source code or screenshots/videos alongside the article). As pages behind login screens cannot be archived, consider creating demo pages which are accessible without login.

	1	2	3	4	5	
subitem not at all important	◯	◯	◯	◯	◯	essential

Does your paper address subitem 5-vi?

Copy and paste relevant sections from the manuscript (include quotes in quotation marks "like this" to indicate direct quotes from your manuscript), or elaborate on this item by providing additional information not in the ms, or briefly explain why the item is not applicable/relevant for your study

5-vii) Access

Access: Describe how participants accessed the application, in what setting/context, if they had to pay (or were paid) or not, whether they had to be a member of specific group. If known, describe how participants obtained “access to the platform and Internet” [1]. To ensure access for editors/reviewers/readers, consider to provide a “backdoor” login account or demo mode for reviewers/readers to explore the application (also important for archiving purposes, see vi).

	1	2	3	4	5	
subitem not at all important	◯	◯	◯	◯	◯	essential

Does your paper address subitem 5-vii? *

Copy and paste relevant sections from the manuscript (include quotes in quotation marks "like this" to indicate direct quotes from your manuscript), or elaborate on this item by providing additional information not in the ms, or briefly explain why the item is not applicable/relevant for your study

The manuscript does describe how participants accessed the application:

"Four Australian high schools - two Anglican girls schools (referred to as Girls School A and Girls School B), a Catholic boys school (referred to as Boys School), and a Jewish co-educational school (referred to as Co-ed School) agreed to participate in the study. Students were in Years 7 through to 12 which in the Australian educative system are the final 6 years before being eligible to go to university. These four schools were amongst the highest in terms of socioeconomic status compared to other schools in Australia. Students were included in the study only if they completed the relevant consent forms. This study was approved by the UNSW Human Research Ethics Advisory Committee (Ethics Approval number 2011-7-35)."

"Between the dates of January 2012 and March 2012, 20 schools were invited to participate in this study, of which four agreed. Prior to study commencement, the lead author met with senior school staff and welfare officers to convey the requirements of the study and to instruct teachers in the study methodology. Parental and student self-consent were obtained for all participants under the age of 16 years and student self-consent only was obtained for participants aged 16 years and over. The year group designated to participate in the study was chosen by the school and no exclusion criteria were applied.

Teachers were instructed to explain to students that they were participating in a study designed to examine the way in which websites affect how young people view the world. Teachers were also required to provide a 5 minute discussion on this topic prior to the beginning of the study and were asked not to mention that the study included a positive psychology intervention. Teachers were responsible for handing out workbooks, providing students with access to relevant websites, and managing students' behavior during class time. Teachers were unaware as to which classes had been allocated to the intervention or control conditions. Students had no face-to-face contact with any of the researchers prior to, during, or following the study.

Students were randomized by blocks (classes) using an Excel random number generator to allocate them to either the Bite Back or control conditions by an independent researcher not associated with the data collection. Equivalent numbers of students were allocated to each condition across each year group and for each school (e.g. Year 9 at the Boys School). In cases where there were uneven numbers of blocks, students were assigned to the Bite Back condition. Students completed an online battery of self-report questionnaires twice during class time: pre- and post-intervention.

All schools were instructed to use the workbook over a six-week period. However, two schools (the Boys School and the Co-ed School) completed the workbook over a four week period due to class-time constraints. Teachers were told that a total of six hours of face-to-face time was required from students over a period of four to six weeks. After each session, students in both the control and Bite Back conditions were asked to email completed sections [illegible]

5-viii) Mode of delivery, features/functionalities/components of the intervention and comparator, and the theoretical framework

Describe mode of delivery, features/functionalities/components of the intervention and comparator, and the theoretical framework [6] used to design them (instructional strategy [1],

behaviour change techniques, persuasive features, etc., see e.g., [7, 8] for terminology). This includes an in-depth description of the content (including where it is coming from and who developed it) [1]," whether [and how] it is tailored to individual circumstances and allows users to track their progress and receive feedback" [6]. This also includes a description of communication delivery channels and – if computer-mediated communication is a component – whether communication was synchronous or asynchronous [6]. It also includes information on presentation strategies [1], including page design principles, average amount of text on pages, presence of hyperlinks to other resources, etc. [1].

	1	2	3	4	5	
subitem not at all important	○	○	○	○	○	essential

Does your paper address subitem 5-viii? *

Copy and paste relevant sections from the manuscript (include quotes in quotation marks "like this" to indicate direct quotes from your manuscript), or elaborate on this item by providing additional information not in the ms, or briefly explain why the item is not applicable/relevant for your study

Yes, the mode of delivery, features/functionalities/components of the intervention and comparator and theoretical framework are described:

"In this regard, positive psychology, the scientific study of happiness, wellbeing, and flourishing, may offer an alternative to conventional prevention programs for youth. Positive psychology can be conceptualized as a range of behavioral, attitudinal and emotional domains that are important for improving wellbeing. These include: developing healthy social relationships, becoming more optimism, finding 'meaning' and practicing mindfulness. Evaluations of non-school-based programs have reported on the benefits of engaging in positive psychology exercises. For example, interventions that increase hope have been shown to predict lower illicit substance use, lower levels of depression, anxiety and hostility, less behavioral problems, and higher academic performance in adolescents [7, 8].

Manicavasagar et al., conducted a feasibility study of a community-based online positive psychological program 'Bite Back' for adolescents aged between 12-18 year [9, 10]. Bite Back comprised information and interactive activities relating to nine domains: gratitude, optimism, flow, meaning, hope, mindfulness, character strengths, healthy lifestyle, and positive relationships. Participants in both the Bite Back condition and a control condition were encouraged to regularly use their respective websites over a six-week period. Symptom measures of depression, anxiety and stress, and wellbeing were compared within and between conditions and at baseline and post-intervention. At the start of the intervention, participants reported low levels of psychopathology, as was expected in a non-clinical sample. Results demonstrated significant decreases in symptom scores and increased scores on wellbeing post-intervention for the Bite Back relative to the control condition. It was reported that when participants with high levels of engagement were examined in isolation, the findings were even stronger suggesting that increased usage was related to greater benefits. Furthermore, qualitative analyses indicated that Bite Back was well received by participants allocated to this condition. It is notable that participants using this online positive psychology program could freely engage with the material at their own pace and in their own time. However, the open-access online format of this program meant that the researchers were unable to examine how participants navigated the website and whether they were exposed to the full range of material and information about positive psychology."

"The Positive Psychology Condition (Bite Back). Bite Back was developed by the Black Dog Institute to improve the wellbeing and happiness of young Australians between the ages of 12 and 18

[illegible]
years. Key objectives of this program are to encourage young people to work to their full potential, become more fully engaged in all aspects of their lives and, ultimately, to build resilience. The program comprises a range of interactive activities including making gratitude entries, mindfulness meditations, describing personal stories, and a mindfulness exercise involving taking photos. Online interactive exercises are designed to encourage generalization of online activities to the 'real world', so that young people benefit from implementing the central tenets of positive psychology into their daily lives. Also included on the site is information about nine positive psychology domains and ways to engage with them outside of the website - Gratitude, Optimism, Flow, Meaning, Hope, Mindfulness, Character Strengths, Healthy Lifestyle and Positive Relationships. The nine domains were based on research that suggested they were important in improving wellbeing [17]. Users are able to comment on activities (such as submitted photos or stories by other users) and are encouraged to generate an anonymous profile thereby enabling participation without fear of stigma or judgment. The website is pre-moderated to ensure that anti-social or concerning behavior is addressed prior to possible posting on the website. For the purposes of the study, a workbook was developed to guide students through the positive psychology exercises on the website and to pose reflective questions about positive psychology. This ensured that participants had sufficient material to use during the course of the program and encouraged users to engage with several domains within the website. Participants were required to record all comments and answers to questions in their workbooks.

The Control Condition. Participants assigned to the control condition used a workbook that was structured in a similar format to that of the Bite Back workbook but with questions and links to non-psychology websites: Australian Broadcasting Corporation, Australian Youth Climate Coalition, the Natural Resource Defense Council, World Wildlife Fund, Wikipedia, Internet Movie Database, and World Youth News. Questions such as 'Do you read online news websites in your own time? If yes, which ones?'; 'Do you think the news makes a difference to how you live in your daily life? Why?'; and 'Write a summary of what the article was about' were designed to encourage users to engage with these websites and record their comments and answers to questions in their workbooks. "

5-ix) Describe use parameters

Describe use parameters (e.g., intended "doses" and optimal timing for use). Clarify what instructions or recommendations were given to the user, e.g., regarding timing, frequency, heaviness of use, if any, or was the intervention used ad libitum.

	1	2	3	4	5	
subitem not at all important	○	○	○	○	○	essential

Does your paper address subitem 5-ix?

Copy and paste relevant sections from the manuscript (include quotes in quotation marks "like this" to indicate direct quotes from your manuscript), or elaborate on this item by providing additional information not in the ms, or briefly explain why the item is not applicable/relevant for your study

5-x) Clarify the level of human involvement

Clarify the level of human involvement (care providers or health professionals, also technical assistance) in the e-intervention or as co-intervention (detail number and expertise of professionals involved, if any, as well as “type of assistance offered, the timing and frequency of the support, how it is initiated, and the medium by which the assistance is delivered”. It may be necessary to distinguish between the level of human involvement required for the trial, and the level of human involvement required for a routine application outside of a RCT setting (discuss under item 21 – generalizability).

	1	2	3	4	5	
subitem not at all important	◯	◯	◯	◯	◯	essential

Does your paper address subitem 5-x?

Copy and paste relevant sections from the manuscript (include quotes in quotation marks "like this" to indicate direct quotes from your manuscript), or elaborate on this item by providing additional information not in the ms, or briefly explain why the item is not applicable/relevant for your study

5-xi) Report any prompts/reminders used

Report any prompts/reminders used: Clarify if there were prompts (letters, emails, phone calls, SMS) to use the application, what triggered them, frequency etc. It may be necessary to distinguish between the level of prompts/reminders required for the trial, and the level of prompts/reminders for a routine application outside of a RCT setting (discuss under item 21 – generalizability).

	1	2	3	4	5	
subitem not at all important	◯	◯	◯	◯	◯	essential

Does your paper address subitem 5-xi? *

Copy and paste relevant sections from the manuscript (include quotes in quotation marks "like this" to indicate direct quotes from your manuscript), or elaborate on this item by providing additional information not in the ms, or briefly explain why the item is not applicable/relevant for your study

Yes, the use of prompts/reminders were reported. In our study, no prompts or reminders were employed but participants were required to email their weekly workbooks to the researchers which served to increase engagement and compliance. See details below:

"All schools were instructed to use the workbook over a six-week period. However, two schools (the Boys School and the Co-ed School) completed the workbook over a four week period due to class-time constraints. Teachers were told that a total of six hours of face-to-face time was required from students over a period of four to six weeks. After each session, students in both the control and Bite Back conditions were asked to email completed sections of their workbooks to the researchers. This strategy was employed to encourage engagement and increase compliance. It also served as an indicator of student adherence to the research tasks. Students were advised that their teachers did not have access to their workbooks and completed questionnaires."

5-xii) Describe any co-interventions (incl. training/support)

Describe any co-interventions (incl. training/support): Clearly state any interventions that are provided in addition to the targeted eHealth intervention, as ehealth intervention may not be designed as stand-alone intervention. This includes training sessions and support [1]. It may be necessary to distinguish between the level of training required for the trial, and the level of training for a routine application outside of a RCT setting (discuss under item 21 – generalizability.

	1	2	3	4	5	
subitem not at all important	◯	◯	◯	◯	◯	essential

Does your paper address subitem 5-xii? *

Copy and paste relevant sections from the manuscript (include quotes in quotation marks "like this" to indicate direct quotes from your manuscript), or elaborate on this item by providing additional information not in the ms, or briefly explain why the item is not applicable/relevant for your study

There were no co-interventions

6a) Completely defined pre-specified primary and secondary outcome measures, including how and when they were assessed

Does your paper address CONSORT subitem 6a? *

Copy and paste relevant sections from the manuscript (include quotes in quotation marks "like this" to indicate direct quotes from your manuscript), or elaborate on this item by providing additional information not in the ms, or briefly explain why the item is not applicable/relevant for your study

As this was a feasibility study there were no primary or secondary outcome measures.

6a-i) Online questionnaires: describe if they were validated for online use and apply CHERRIES items to describe how the questionnaires were designed/deployed

If outcomes were obtained through online questionnaires, describe if they were validated for online use and apply CHERRIES items to describe how the questionnaires were designed/deployed [9].

	1	2	3	4	5	
subitem not at all important	○	○	○	○	○	essential

Does your paper address subitem 6a-i?

Copy and paste relevant sections from manuscript text

6a-ii) Describe whether and how “use” (including intensity of use/dosage) was defined/measured/monitored

Describe whether and how “use” (including intensity of use/dosage) was defined/measured/monitored (logins, logfile analysis, etc.). Use/adoption metrics are important process outcomes that should be reported in any ehealth trial.

	1	2	3	4	5	
subitem not at all important	○	○	○	○	○	essential

Does your paper address subitem 6a-ii?

Copy and paste relevant sections from manuscript text

6a-iii) Describe whether, how, and when qualitative feedback from participants was obtained

Describe whether, how, and when qualitative feedback from participants was obtained (e.g., through emails, feedback forms, interviews, focus groups).

	1	2	3	4	5	
subitem not at all important	○	○	○	○	○	essential

Does your paper address subitem 6a-iii?
Copy and paste relevant sections from manuscript text

6b) Any changes to trial outcomes after the trial commenced, with reasons

Does your paper address CONSORT subitem 6b? *
Copy and paste relevant sections from the manuscript (include quotes in quotation marks "like this" to indicate direct quotes from your manuscript), or elaborate on this item by providing additional information not in the ms, or briefly explain why the item is not applicable/relevant for your study

There were no changes to trial outcomes after the trial commenced.

7a) How sample size was determined

NPT: When applicable, details of whether and how the clustering by care provides or centers was addressed

7a-i) Describe whether and how expected attrition was taken into account when calculating the sample size
Describe whether and how expected attrition was taken into account when calculating the sample size.

	1	2	3	4	5	
subitem not at all important	○	○	○	○	○	essential

Does your paper address subitem 7a-i?
Copy and paste relevant sections from manuscript title (include quotes in quotation marks "like this" to indicate direct quotes from your manuscript), or elaborate on this item by providing additional information not in the ms, or briefly explain why the item is not applicable/relevant for your study

7b) When applicable, explanation of any interim analyses and stopping guidelines

Does your paper address CONSORT subitem 7b? *

Copy and paste relevant sections from the manuscript (include quotes in quotation marks "like this" to indicate direct quotes from your manuscript), or elaborate on this item by providing additional information not in the ms, or briefly explain why the item is not applicable/relevant for your study

There were no interim analyses or stopping guidelines due to the feasibility study nature of our study.

8a) Method used to generate the random allocation sequence

NPT: When applicable, how care providers were allocated to each trial group

Does your paper address CONSORT subitem 8a? *

Copy and paste relevant sections from the manuscript (include quotes in quotation marks "like this" to indicate direct quotes from your manuscript), or elaborate on this item by providing additional information not in the ms, or briefly explain why the item is not applicable/relevant for your study

This was described. See below:

"Students were randomized by blocks (classes) using an Excel random number generator to allocate them to either the Bite Back or control conditions by an independent researcher not associated with the data collection. Equivalent numbers of students were allocated to each condition across each year group and for each school (e.g. Year 9 at the Boys School). In cases where there were uneven numbers of blocks, students were assigned to the Bite Back condition. Students completed an online battery of self-report questionnaires twice during class time: pre- and post-intervention."

8b) Type of randomisation; details of any restriction (such as blocking and block size)

Does your paper address CONSORT subitem 8b? *

Copy and paste relevant sections from the manuscript (include quotes in quotation marks "like this" to indicate direct quotes from your manuscript), or elaborate on this item by providing additional information not in the ms, or briefly explain why the item is not applicable/relevant for your study

The randomisation process is described in the manuscript. See below:

"Students were randomized by blocks (classes) using an Excel random number generator to allocate them to either the Bite Back or control conditions by an independent researcher not associated with the data collection. Equivalent numbers of students were allocated to each condition across each year group and for each school (e.g. Year 9 at the Boys School). In cases where there were uneven numbers of blocks, students were assigned to the Bite Back condition. Students completed an online battery of self-report questionnaires twice during class time: pre- and post-intervention."

9) Mechanism used to implement the random allocation sequence (such as sequentially numbered containers), describing any steps taken to conceal the sequence until interventions were assigned

Does your paper address CONSORT subitem 9? *

Copy and paste relevant sections from the manuscript (include quotes in quotation marks "like this" to indicate direct quotes from your manuscript), or elaborate on this item by providing additional information not in the ms, or briefly explain why the item is not applicable/relevant for your study

This is described. See the quoted text:

"Students were randomized by blocks (classes) using an Excel random number generator to allocate them to either the Bite Back or control conditions by an independent researcher not associated with the data collection. Equivalent numbers of students were allocated to each condition across each year group and for each school (e.g. Year 9 at the Boys School). In cases where there were uneven numbers of blocks, students were assigned to the Bite Back condition. Students completed an online battery of self-report questionnaires twice during class time: pre- and post-intervention."

10) Who generated the random allocation sequence, who enrolled participants, and who assigned participants to interventions

Does your paper address CONSORT subitem 10? *

Copy and paste relevant sections from the manuscript (include quotes in quotation marks "like this" to indicate direct quotes from your manuscript), or elaborate on this item by providing additional information not in the ms, or briefly explain why the item is not applicable/relevant for your study

This is outlined in the method section. See below:

"Students were randomized by blocks (classes) using an Excel random number generator to allocate them to either the Bite Back or control conditions by an independent researcher not associated with the data collection. Equivalent numbers of students were allocated to each condition across each year group and for each school (e.g. Year 9 at the Boys School). In cases where there were uneven numbers of blocks, students were assigned to the Bite Back condition. Students completed an online battery of self-report questionnaires twice during class time: pre- and post-intervention."

11a) If done, who was blinded after assignment to interventions (for example, participants, care providers, those assessing outcomes) and how

NPT: Whether or not administering co-interventions were blinded to group assignment

11a-i) Specify who was blinded, and who wasn't

Specify who was blinded, and who wasn't. Usually, in web-based trials it is not possible to blind the participants [1, 3] (this should be clearly acknowledged), but it may be possible to blind outcome assessors, those doing data analysis or those administering co-interventions (if any).

	1	2	3	4	5	
subitem not at all important	○	○	○	○	○	essential

Does your paper address subitem 11a-i? *

Copy and paste relevant sections from the manuscript (include quotes in quotation marks "like this" to indicate direct quotes from your manuscript), or elaborate on this item by providing additional information not in the ms, or briefly explain why the item is not applicable/relevant for your study

Blinding was addressed by telling participants the study sought to understand how websites affect the way young people view the world. See a description below:

"Teachers were instructed to explain to students that they were participating in a study designed to examine the way in which websites affect how young people view the world. Teachers were also required to provide a 5 minute discussion on this topic prior to the beginning of the study and were asked not to mention that the study included a positive psychology intervention. Teachers were responsible for handing out workbooks, providing students with access to relevant websites, and managing students' behavior during class time. Teachers were unaware as to which classes had been allocated to the intervention or control conditions. Students had no face-to-face contact with any of the researchers prior to, during, or following the study."

11a-ii) Discuss e.g., whether participants knew which intervention was the "intervention of interest" and which one was the "comparator"

Informed consent procedures (4a-ii) can create biases and certain expectations - discuss e.g., whether participants knew which intervention was the "intervention of interest" and which one was the "comparator".

	1	2	3	4	5	
subitem not at all important	○	○	○	○	○	essential

Does your paper address subitem 11a-ii?

Copy and paste relevant sections from the manuscript (include quotes in quotation marks "like this" to indicate direct quotes from your manuscript), or elaborate on this item by providing additional information not in the ms, or briefly explain why the item is not applicable/relevant for your study

11b) If relevant, description of the similarity of interventions

(this item is usually not relevant for ehealth trials as it refers to similarity of a placebo or sham intervention to a active medication/intervention)

Does your paper address CONSORT subitem 11b? *

Copy and paste relevant sections from the manuscript (include quotes in quotation marks "like this" to indicate direct quotes from your manuscript), or elaborate on this item by providing additional information not in the ms, or briefly explain why the item is not applicable/relevant for your study

Attempts were made to keep the two interventions as similar to one another as possible. See below:

"The Positive Psychology Condition (Bite Back). Bite Back was developed by the Black Dog Institute to improve the wellbeing and happiness of young Australians between the ages of 12 and 18 years. Key objectives of this program are to encourage young people to work to their full potential, become more fully engaged in all aspects of their lives and, ultimately, to build resilience. The program comprises a range of interactive activities including making gratitude entries, mindfulness meditations, describing personal stories, and a mindfulness exercise involving taking photos. Online interactive exercises are designed to encourage generalization of online activities to the 'real world', so that young people benefit from implementing the central tenets of positive psychology into their daily lives. Also included on the site is information about nine positive psychology domains and ways to engage with them outside of the website - Gratitude, Optimism, Flow, Meaning, Hope, Mindfulness, Character Strengths, Healthy Lifestyle and Positive Relationships. The nine domains were based on research that suggested they were important in improving wellbeing [17]. Users are able to comment on activities (such as submitted photos or stories by other users) and are encouraged to generate an anonymous profile thereby enabling participation without fear of stigma or judgment. The website is pre-moderated to ensure that anti-social or concerning behavior is addressed prior to possible posting on the website. For the purposes of the study, a workbook was developed to guide students through the positive psychology exercises on the website and to pose reflective questions about positive psychology. This ensured that participants had sufficient material to use during the course of the program and encouraged users to engage with several domains within the website. Participants were required to record all comments and answers to questions in their workbooks.

The Control Condition. Participants assigned to the control condition used a workbook that was structured in a similar format to that of the Bite Back workbook but with questions and links to non-psychology websites: Australian Broadcasting Corporation, Australian Youth Climate Coalition, the Natural Resource Defense Council, World Wildlife Fund, Wikipedia, Internet Movie Database, and World Youth News. Questions such as 'Do you read online news websites in your own time? If yes, which ones?'; 'Do you think the news makes a difference to how you live in your daily life? Why?'; and 'Write a summary of what the article was about' were designed to encourage users to engage with these websites and record their comments and answers to questions in their workbooks. "

12a) Statistical methods used to compare groups for primary and secondary outcomes

NPT: When applicable, details of whether and how the clustering by care providers or centers was addressed

Does your paper address CONSORT subitem 12a? *

Copy and paste relevant sections from the manuscript (include quotes in quotation marks "like this" to indicate direct quotes from your manuscript), or elaborate on this item by providing additional information not in the ms, or briefly explain why the item is not applicable/relevant for your study

This was outlined in the manuscript:

"Data was analyzed using SPSS version 22 [26]. Independent samples t-tests were used to examine group differences at baseline. Multilevel linear modelling was used to examine the effectiveness of the intervention in reducing depression, anxiety and stress, and increasing life satisfaction and wellbeing. This type of model represents an intention-to-treat analysis under the missing-at-random assumption. Specifically, the effect of time (post- vs. pre-test) was estimated using a repeated measures mixed effects model, which also accounted for clustering of students within schools using a random intercept for each school. An unstructured variance-covariance structure was assumed for both the repeated effect of time and the random effect of school. Degrees of freedom were estimated using Satterthwaite's correction [27]."

12a-i) Imputation techniques to deal with attrition / missing values

Imputation techniques to deal with attrition / missing values: Not all participants will use the intervention/comparator as intended and attrition is typically high in ehealth trials. Specify how participants who did not use the application or dropped out from the trial were treated in the statistical analysis (a complete case analysis is strongly discouraged, and simple imputation techniques such as LOCF may also be problematic [4]).

	1	2	3	4	5	
subitem not at all important	○	○	○	○	○	essential

Does your paper address subitem 12a-i? *

Copy and paste relevant sections from the manuscript (include quotes in quotation marks "like this" to indicate direct quotes from your manuscript), or elaborate on this item by providing additional information not in the ms, or briefly explain why the item is not applicable/relevant for your study

A multilevel mixed models was used which imputes missing data as part of the analysis strategy.

12b) Methods for additional analyses, such as subgroup analyses and adjusted analyses

Does your paper address CONSORT subitem 12b? *

Copy and paste relevant sections from the manuscript (include quotes in quotation marks "like this" to indicate direct quotes from your manuscript), or elaborate on this item by providing additional information not in the ms, or briefly explain why the item is not applicable/relevant for your study

A second universal effects analysis was completed on the data controlling for baseline symptom severity. See an example of the depression subscale below. The same was used on the other subscales.

"Given that the baseline levels of psychopathology were low, the impact of the website on those reporting high levels of symptoms at the start of the program was also examined. The analysis was repeated amongst participants in the mild to extremely high categories of DASS depression baseline scores (n=226). No significant differences between the control and the Bite Back conditions were found. To examine if the level of engagement modified the results, the analysis was repeated to compare participants with low versus high engagement with the workbook sections. No significant differences were found between the groups."

X26) REB/IRB Approval and Ethical Considerations [recommended as subheading under "Methods"] (not a CONSORT item)

X26-i) Comment on ethics committee approval

	1	2	3	4	5	
subitem not at all important	○	○	○	○	○	essential

Does your paper address subitem X26-i?

Copy and paste relevant sections from the manuscript (include quotes in quotation marks "like this" to indicate direct quotes from your manuscript), or elaborate on this item by providing additional information not in the ms, or briefly explain why the item is not applicable/relevant for your study

x26-ii) Outline informed consent procedures

Outline informed consent procedures e.g., if consent was obtained offline or online (how? Checkbox, etc.?), and what information was provided (see 4a-ii). See [6] for some items to be included in informed consent documents.

	1	2	3	4	5	
subitem not at all important	○	○	○	○	○	essential

Does your paper address subitem X26-ii?

Copy and paste relevant sections from the manuscript (include quotes in quotation marks "like this" to indicate direct quotes from your manuscript), or elaborate on this item by providing additional

information not in the ms, or briefly explain why the item is not applicable/relevant for your study

X26-iii) Safety and security procedures

Safety and security procedures, incl. privacy considerations, and any steps taken to reduce the likelihood or detection of harm (e.g., education and training, availability of a hotline)

	1	2	3	4	5	
subitem not at all important	○	○	○	○	○	essential

Does your paper address subitem X26-iii?

Copy and paste relevant sections from the manuscript (include quotes in quotation marks "like this" to indicate direct quotes from your manuscript), or elaborate on this item by providing additional information not in the ms, or briefly explain why the item is not applicable/relevant for your study

RESULTS

13a) For each group, the numbers of participants who were randomly assigned, received intended treatment, and were analysed for the primary outcome

NPT: The number of care providers or centers performing the intervention in each group and the number of patients treated by each care provider in each center

Does your paper address CONSORT subitem 13a? *

Copy and paste relevant sections from the manuscript (include quotes in quotation marks "like this" to indicate direct quotes from your manuscript), or elaborate on this item by providing additional information not in the ms, or briefly explain why the item is not applicable/relevant for your study

Yes, the CONSORT flowchart is outlined in Figure 1. In addition, there is a description of the main parts as follows:

"Figure 1 demonstrates the flow of participants during the course of the study. Four schools provided at total of 572 students across years 7 to 12 which were randomly allocated through classroom blocks to either the control or Bite Back conditions. Of those allocated, 338 students completed a battery of online questionnaires at baseline and were considered to be enrolled in the study. From the 234 students who did not complete the baseline questionnaire, 110 encountered technical difficulties that prevented them from being able to access the questionnaire successfully. The other 124 either chose not to fill out the questionnaire or were absent. Of the four schools that were enrolled, one withdrew from the study (Girls School B) and one ceased to participate because the teacher involved in its implementation went on extended leave (Co-ed School). Baseline characteristics of participants are presented in Table 1."

13b) For each group, losses and exclusions after randomisation, together with reasons

Does your paper address CONSORT subitem 13b? (NOTE: Preferably, this is shown in a CONSORT flow diagram) *

Copy and paste relevant sections from the manuscript (include quotes in quotation marks "like this" to indicate direct quotes from your manuscript), or elaborate on this item by providing additional information not in the ms, or briefly explain why the item is not applicable/relevant for your study

Yes, this is described along with the reasons (there is some additional information on this in CONSORT flow chart in Figure 1 of the manuscript):

"Figure 1 demonstrates the flow of participants during the course of the study. Four schools provided at total of 572 students across years 7 to 12 which were randomly allocated through classroom blocks to either the control or Bite Back conditions. Of those allocated, 338 students completed a battery of online questionnaires at baseline and were considered to be enrolled in the study. From the 234 students who did not complete the baseline questionnaire, 110 encountered technical difficulties that prevented them from being able to access the questionnaire successfully. The other 124 either chose not to fill out the questionnaire or were absent. Of the four schools that were enrolled, one withdrew from the study (Girls School B) and one ceased to participate because the teacher involved in its implementation went on extended leave (Co-ed School). Baseline characteristics of participants are presented in Table 1."

13b-i) Attrition diagram

Strongly recommended: An attrition diagram (e.g., proportion of participants still logging in or using the intervention/comparator in each group plotted over time, similar to a survival curve) or other figures or tables demonstrating usage/dose/engagement.

	1	2	3	4	5	
subitem not at all important	○	○	○	○	○	essential

Does your paper address subitem 13b-i?

Copy and paste relevant sections from the manuscript or cite the figure number if applicable (include quotes in quotation marks "like this" to indicate direct quotes from your manuscript), or elaborate on this item by providing additional information not in the ms, or briefly explain why the item is not applicable/relevant for your study

14a) Dates defining the periods of recruitment and follow-up

Does your paper address CONSORT subitem 14a? *

Copy and paste relevant sections from the manuscript (include quotes in quotation marks "like this" to indicate direct quotes from your manuscript), or elaborate on this item by providing additional information not in the ms, or briefly explain why the item is not applicable/relevant for your study

Yes, see below:

"Between the dates of January 2012 and March 2012, 20 schools were invited to participate in this study, of which four agreed. Prior to study commencement, the lead author met with senior school staff and welfare officers to convey the requirements of the study and to instruct teachers in the study methodology. Parental and student self-consent were obtained for all participants under the age of 16 years and student self-consent only was obtained for participants aged 16 years and over. The year group designated to participate in the study was chosen by the school and no exclusion criteria were applied."

14a-i) Indicate if critical “secular events” fell into the study period

Indicate if critical “secular events” fell into the study period, e.g., significant changes in Internet resources available or “changes in computer hardware or Internet delivery resources”

	1	2	3	4	5	
subitem not at all important	○	○	○	○	○	essential

Does your paper address subitem 14a-i?

Copy and paste relevant sections from the manuscript (include quotes in quotation marks "like this" to indicate direct quotes from your manuscript), or elaborate on this item by providing additional information not in the ms, or briefly explain why the item is not applicable/relevant for your study

14b) Why the trial ended or was stopped (early)

Does your paper address CONSORT subitem 14b? *

Copy and paste relevant sections from the manuscript (include quotes in quotation marks "like this" to indicate direct quotes from your manuscript), or elaborate on this item by providing additional information not in the ms, or briefly explain why the item is not applicable/relevant for your study

The trial was stopped once pre and post data was collected. This was the intention. Further it was planned that the program would be trialled in a small group to examine feasibility of implementation and so the sample size used was adequate to address these objectives. Furthermore, it was a study that was conducted to compare the feasibility of using Bite Back in a school context compared to a community study and therefore a similar sample size was required, which was obtained.

15) A table showing baseline demographic and clinical characteristics for each group

NPT: When applicable, a description of care providers (case volume, qualification, expertise, etc.) and centers (volume) in each group

Does your paper address CONSORT subitem 15? *

Copy and paste relevant sections from the manuscript (include quotes in quotation marks "like this" to indicate direct quotes from your manuscript), or elaborate on this item by providing additional information not in the ms, or briefly explain why the item is not applicable/relevant for your study

Yes, see table 1 of the manuscript

15-i) Report demographics associated with digital divide issues

In ehealth trials it is particularly important to report demographics associated with digital divide issues, such as age, education, gender, social-economic status, computer/Internet/ehealth literacy of the participants, if known.

	1	2	3	4	5	
subitem not at all important	○	○	○	○	○	essential

Does your paper address subitem 15-i? *

Copy and paste relevant sections from the manuscript (include quotes in quotation marks "like this" to indicate direct quotes from your manuscript), or elaborate on this item by providing additional information not in the ms, or briefly explain why the item is not applicable/relevant for your study

Yes, see table 1 of the manuscript. In addition, see quote below:

"Four Australian high schools - two Anglican girls schools (referred to as Girls School A and Girls School B), a Catholic boys school (referred to as Boys School), and a Jewish co-educational school (referred to as Co-ed School) agreed to participate in the study. Students were in Years 7 through to 12 which in the Australian educative system are the final 6 years before being eligible to go to university. These four schools were amongst the highest in terms of socioeconomic status compared to other schools in Australia. Students were included in the study only if they completed the relevant consent forms. This study was approved by the UNSW Human Research Ethics Advisory Committee (Ethics Approval number 2011-7-35)."

16) For each group, number of participants (denominator) included in each analysis and whether the analysis was by original assigned groups

16-i) Report multiple "denominators" and provide definitions

Report multiple "denominators" and provide definitions: Report N's (and effect sizes) "across a range of study participation [and use] thresholds" [1], e.g., N exposed, N consented, N used more than x times, N used more than y weeks, N participants "used" the intervention/comparator at specific pre-defined time points of interest (in absolute and relative numbers per group). Always clearly define "use" of the intervention.

	1	2	3	4	5	
subitem not at all important	○	○	○	○	○	essential

Does your paper address subitem 16-i? *

Copy and paste relevant sections from the manuscript (include quotes in quotation marks "like this" to indicate direct quotes from your manuscript), or elaborate on this item by providing additional information not in the ms, or briefly explain why the item is not applicable/relevant for your study

Yes, see table 1 and in addition, the comments from the depression subscale analysis:

"Depression. Results indicated a non-significant interaction between time and condition for DASS-Depression scores, F(1, 199.9)=.42, P=.52. The effect of time alone was significant, F(1, 309.1)=8.10, P=.005, indicating a significant reduction of depression from baseline (M=8.57) to post-intervention (M=6.93) for both conditions but no significant differences between the groups. Given that the baseline levels of psychopathology were low, the impact of the website on those reporting high levels of symptoms at the start of the program was also examined. The analysis was repeated amongst participants in the mild to extremely high categories of DASS depression baseline scores (n=226). No significant differences between the control and the Bite Back conditions were found. To examine if the level of engagement modified the results, the analysis was repeated to compare participants with low versus high engagement with the workbook sections. No significant differences were found between the groups."

16-ii) Primary analysis should be intent-to-treat

Primary analysis should be intent-to-treat, secondary analyses could include comparing only "users", with the appropriate caveats that this is no longer a randomized sample (see 18-i).

	1	2	3	4	5	
subitem not at all important	○	○	○	○	○	essential

Does your paper address subitem 16-ii?

Copy and paste relevant sections from the manuscript (include quotes in quotation marks "like this" to indicate direct quotes from your manuscript), or elaborate on this item by providing additional information not in the ms, or briefly explain why the item is not applicable/relevant for your study

17a) For each primary and secondary outcome, results for each group, and the estimated effect size and its precision (such as 95% confidence interval)

Does your paper address CONSORT subitem 17a? *

Copy and paste relevant sections from the manuscript (include quotes in quotation marks "like this" to indicate direct quotes from your manuscript), or elaborate on this item by providing additional information not in the ms, or briefly explain why the item is not applicable/relevant for your study

Given this was a feasibility study, effect sizes were not included.

17a-i) Presentation of process outcomes such as metrics of use and intensity of use

In addition to primary/secondary (clinical) outcomes, the presentation of process outcomes such as metrics of use and intensity of use (dose, exposure) and their operational definitions is critical. This does not only refer to metrics of attrition (13-b) (often a binary variable), but also to more continuous exposure metrics such as "average session length". These must be accompanied by a technical description how a metric like a "session" is defined (e.g., timeout after idle time) [1] (report under item 6a).

	1	2	3	4	5	
subitem not at all important	○	○	○	○	○	essential

Does your paper address subitem 17a-i?

Copy and paste relevant sections from the manuscript (include quotes in quotation marks "like this" to indicate direct quotes from your manuscript), or elaborate on this item by providing additional information not in the ms, or briefly explain why the item is not applicable/relevant for your study

17b) For binary outcomes, presentation of both absolute and relative effect sizes is recommended

Does your paper address CONSORT subitem 17b? *

Copy and paste relevant sections from the manuscript (include quotes in quotation marks "like this" to indicate direct quotes from your manuscript), or elaborate on this item by providing additional information not in the ms, or briefly explain why the item is not applicable/relevant for your study

As this study was a feasibility study, no effect sizes were reported.

18) Results of any other analyses performed, including subgroup analyses and adjusted analyses, distinguishing pre-specified from exploratory

Does your paper address CONSORT subitem 18? *

Copy and paste relevant sections from the manuscript (include quotes in quotation marks "like this" to indicate direct quotes from your manuscript), or elaborate on this item by providing additional information not in the ms, or briefly explain why the item is not applicable/relevant for your study

A subgroup analysis was completed using the baseline symptom level to dichotomise the groups. See the quote from the depression subscale as an example:

"Depression. Results indicated a non-significant interaction between time and condition for DASS-Depression scores, F(1, 199.9)=.42, P=.52. The effect of time alone was significant, F(1, 309.1)=8.10, P=.005, indicating a significant reduction of depression from baseline (M=8.57) to post-intervention (M=6.93) for both conditions but no significant differences between the groups. Given that the baseline levels of psychopathology were low, the impact of the website on those reporting high levels of symptoms at the start of the program was also examined. The analysis was repeated amongst participants in the mild to extremely high categories of DASS depression baseline scores (n=226). No significant differences between the control and the Bite Back conditions were found. To examine if the level of engagement modified the results, the analysis was repeated to compare participants with low versus high engagement with the workbook sections. No significant differences were found between the groups."

18-i) Subgroup analysis of comparing only users

A subgroup analysis of comparing only users is not uncommon in ehealth trials, but if done, it must be stressed that this is a self-selected sample and no longer an unbiased sample from a randomized trial (see 16-iii).

	1	2	3	4	5	
subitem not at all important	○	○	○	○	○	essential

Does your paper address subitem 18-i?

Copy and paste relevant sections from the manuscript (include quotes in quotation marks "like this" to indicate direct quotes from your manuscript), or elaborate on this item by providing additional information not in the ms, or briefly explain why the item is not applicable/relevant for your study

19) All important harms or unintended effects in each group

(for specific guidance see CONSORT for harms)

Does your paper address CONSORT subitem 19? *

Copy and paste relevant sections from the manuscript (include quotes in quotation marks "like this" to indicate direct quotes from your manuscript), or elaborate on this item by providing additional information not in the ms, or briefly explain why the item is not applicable/relevant for your study

There were no harms or unintended effects in this study.

19-i) Include privacy breaches, technical problems

Include privacy breaches, technical problems. This does not only include physical “harm” to participants, but also incidents such as perceived or real privacy breaches [1], technical problems, and other unexpected/unintended incidents. “Unintended effects” also includes unintended positive effects [2].

	1	2	3	4	5	
subitem not at all important	○	○	○	○	○	essential

Does your paper address subitem 19-i?

Copy and paste relevant sections from the manuscript (include quotes in quotation marks "like this" to indicate direct quotes from your manuscript), or elaborate on this item by providing additional information not in the ms, or briefly explain why the item is not applicable/relevant for your study

19-ii) Include qualitative feedback from participants or observations from staff/researchers

Include qualitative feedback from participants or observations from staff/researchers, if available, on strengths and shortcomings of the application, especially if they point to unintended/unexpected effects or uses. This includes (if available) reasons for why people did or did not use the application as intended by the developers.

	1	2	3	4	5	
subitem not at all important	○	○	○	○	○	essential

Does your paper address subitem 19-ii?

Copy and paste relevant sections from the manuscript (include quotes in quotation marks "like this" to indicate direct quotes from your manuscript), or elaborate on this item by providing additional information not in the ms, or briefly explain why the item is not applicable/relevant for your study

DISCUSSION

22) Interpretation consistent with results, balancing benefits and harms, and considering other relevant evidence

NPT: In addition, take into account the choice of the comparator, lack of or partial blinding, and unequal expertise of care providers or centers in each group

22-i) Restate study questions and summarize the answers suggested by the data, starting with primary outcomes and process outcomes (use)

Restate study questions and summarize the answers suggested by the data, starting with primary outcomes and process outcomes (use).

	1	2	3	4	5	
subitem not at all important	○	○	○	○	○	essential

Does your paper address subitem 22-i? *

Copy and paste relevant sections from the manuscript (include quotes in quotation marks "like this" to indicate direct quotes from your manuscript), or elaborate on this item by providing additional information not in the ms, or briefly explain why the item is not applicable/relevant for your study

This was completed in the first paragraph of the discussion:

"The aim of the current study was to evaluate the feasibility and effectiveness of the Bite Back program when delivered to adolescents in a classroom environment. Results from this study indicated that there was a reduction in depression, stress and total symptom scores from baseline to post-intervention for the control and Bite Back conditions. However, participants from the two conditions did not significantly differ in their change in any of these variables. On the life satisfaction scale, participants reported an overall reduction in scores from baseline to post-intervention, although the differences were again not significantly different across the control and Bite Back conditions. For the flourishing scale, there was a significant difference between the control group and Bite Back across the two time points, favoring control. However, the control condition started the intervention with lower scores and returned to the same level as the Bite Back condition post-intervention, so it is difficult to establish if this was a case of regression to the mean or if there was a more positive impact of the control condition compared to Bite Back. The principle conclusion that can be drawn from these results is that there was no significant benefit of participating in the Bite Back condition

22-ii) Highlight unanswered new questions, suggest future research

Highlight unanswered new questions, suggest future research.

	1	2	3	4	5	
subitem not at all important	○	○	○	○	○	essential

Does your paper address subitem 22-ii?

Copy and paste relevant sections from the manuscript (include quotes in quotation marks "like this" to indicate direct quotes from your manuscript), or elaborate on this item by providing additional information not in the ms, or briefly explain why the item is not applicable/relevant for your study

20) Trial limitations, addressing sources of potential bias, imprecision, and, if relevant, multiplicity of analyses

20-i) Typical limitations in ehealth trials

Typical limitations in ehealth trials: Participants in ehealth trials are rarely blinded. Ehealth trials often look at a multiplicity of outcomes, increasing risk for a Type I error. Discuss biases due to non-use of the intervention/usability issues, biases through informed consent procedures, unexpected events.

	1	2	3	4	5	
subitem not at all important	◯	◯	◯	◯	◯	essential

Does your paper address subitem 20-i? *

Copy and paste relevant sections from the manuscript (include quotes in quotation marks "like this" to indicate direct quotes from your manuscript), or elaborate on this item by providing additional information not in the ms, or briefly explain why the item is not applicable/relevant for your study

The limitations, sources of potential bias and imprecisions were discussed. See below:

"The findings of this study should be interpreted in light of its limitations. The sample used in this study was relatively heterogeneous sample with participants coming from schools that were both co-ed and single gender, and reflected different religious denominations and philosophies. There were relatively few checks on how the program was presented across the various schools and classrooms. This could be both a strength and limitation of the study, as it more closely replicated real-life applications of positive psychology in the school environment.

The sample size was relatively small although this is usual in feasibility type research as it provides data for further large-scale studies. The study was also limited by a lack of long-term follow-up data, although it is common for the strongest reduction in symptoms to be observed at post-intervention and so it is likely that significant differences would not have appeared at a later time point."

21) Generalisability (external validity, applicability) of the trial findings

NPT: External validity of the trial findings according to the intervention, comparators, patients, and care providers or centers involved in the trial

21-i) Generalizability to other populations

Generalizability to other populations: In particular, discuss generalizability to a general Internet population, outside of a RCT setting, and general patient population, including applicability of the study results for other organizations

	1	2	3	4	5	
subitem not at all important	◯	◯	◯	◯	◯	essential

Does your paper address subitem 21-i?

Copy and paste relevant sections from the manuscript (include quotes in quotation marks "like this" to indicate direct quotes from your manuscript), or elaborate on this item by providing additional information not in the ms, or briefly explain why the item is not applicable/relevant for your study

21-ii) Discuss if there were elements in the RCT that would be different in a routine application setting

Discuss if there were elements in the RCT that would be different in a routine application setting (e.g., prompts/reminders, more human involvement, training sessions or other co-interventions) and what impact the omission of these elements could have on use, adoption, or outcomes if the intervention is applied outside of a RCT setting.

	1	2	3	4	5	
subitem not at all important	○	○	○	○	○	essential

Does your paper address subitem 21-ii?

Copy and paste relevant sections from the manuscript (include quotes in quotation marks "like this" to indicate direct quotes from your manuscript), or elaborate on this item by providing additional information not in the ms, or briefly explain why the item is not applicable/relevant for your study

OTHER INFORMATION

23) Registration number and name of trial registry

Does your paper address CONSORT subitem 23? *

Copy and paste relevant sections from the manuscript (include quotes in quotation marks "like this" to indicate direct quotes from your manuscript), or elaborate on this item by providing additional information not in the ms, or briefly explain why the item is not applicable/relevant for your study

Trial registration number: ACTRN1261200057831; https://www.anzctr.org.au/Trial/Registration/TrialReview.aspx?id=362489 (Archived by Webcite at http://www.webcitation.org/6NXmjwfAy)

24) Where the full trial protocol can be accessed, if available

Does your paper address CONSORT subitem 24? *

Cite a Multimedia Appendix, other reference, or copy and paste relevant sections from the manuscript (include quotes in quotation marks "like this" to indicate direct quotes from your manuscript), or elaborate on this item by providing additional information not in the ms, or briefly explain why the item is not applicable/relevant for your study

As this was a feasibility study, the trial protocol was not published prior to the study commencement.

25) Sources of funding and other support (such as supply of drugs), role of funders

Does your paper address CONSORT subitem 25? *

Copy and paste relevant sections from the manuscript (include quotes in quotation marks "like this" to indicate direct quotes from your manuscript), or elaborate on this item by providing additional information not in the ms, or briefly explain why the item is not applicable/relevant for your study

This was declared. See the following quote:

"This study was conducted with the support of a research grant from the Department of Health and Ageing. This work was completed at the Black Dog Institute at the University of New South Wales. We are also grateful to Professor Felicia Huppert for advice on the methodology to this study and to Jacqui Wallace and Nic Newling for technical assistance in running this trial. PB is [illegible]

X27) Conflicts of Interest (not a CONSORT item)

X27-i) State the relation of the study team towards the system being evaluated

In addition to the usual declaration of interests (financial or otherwise), also state the relation of the study team towards the system being evaluated, i.e., state if the authors/evaluators are distinct from or identical with the developers/sponsors of the intervention.

	1	2	3	4	5	
subitem not at all important	○	○	○	○	○	essential

Does your paper address subitem X27-i?

Copy and paste relevant sections from the manuscript (include quotes in quotation marks "like this" to indicate direct quotes from your manuscript), or elaborate on this item by providing additional information not in the ms, or briefly explain why the item is not applicable/relevant for your study

About the CONSORT EHEALTH checklist

As a result of using this checklist, did you make changes in your manuscript? *

- ( ) yes, major changes
- (•) yes, minor changes
- ( ) no

What were the most important changes you made as a result of using this checklist?

We tried to ensure all the points required were addressed prior to writing the manuscript.

How much time did you spend on going through the checklist INCLUDING making changes in your manuscript *

30 hours

As a result of using this checklist, do you think your manuscript has improved? *

- (•) yes
- ( ) no
- ( ) Other:

Would you like to become involved in the CONSORT EHEALTH group?

This would involve for example becoming involved in participating in a workshop and writing an "Explanation and Elaboration" document

- ( ) yes
- (•) no
- ( ) Other:

Any other comments or questions on CONSORT EHEALTH

STOP - Save this form as PDF before you click submit

To generate a record that you filled in this form, we recommend to generate a PDF of this page (on a Mac, simply select "print" and then select "print as PDF") before you submit it.

When you submit your (revised) paper to JMIR, please upload the PDF as supplementary file.

Don't worry if some text in the textboxes is cut off, as we still have the complete information in our database. Thank you!

Final step: Click submit !

Click submit so we have your answers in our database!

Submit

Never submit passwords through Google Forms.

Powered by

This content is neither created nor endorsed by Google.

Report Abuse - Terms of Service - Additional Terms
